# New Optically Active *tert*-Butylarylthiophosphinic Acids and Their Selenium Analogues as the Potential Synthons of Supramolecular Organometallic Complexes: Syntheses and Crystallographic Structure Determination

**DOI:** 10.3390/molecules28114298

**Published:** 2023-05-24

**Authors:** Jarosław Błaszczyk, Bogdan Bujnicki, Patrycja Pokora-Sobczak, Grażyna Mielniczak, Lesław Sieroń, Piotr Kiełbasiński, Józef Drabowicz

**Affiliations:** 1 Division of Organic Chemistry, Centre of Molecular and Macromolecular Studies, Polish Academy of Sciences, Sienkiewicza 112, 90-363 Łódź, Poland; bogdan.bujnicki@cbmm.lodz.pl (B.B.); patrycja.pokora-sobczak@cbmm.lodz.pl (P.P.-S.); grazyna.mielniczak@cbmm.lodz.pl (G.M.); piotr.kielbasinski@cbmm.lodz.pl (P.K.); 2 Institute of General and Ecological Chemistry, Lodz University of Technology, Żeromskiego 116, 90-924 Łódź, Poland; leslaw.sieron@p.lodz.pl; 3 Institute of Chemistry, Jan Długosz University in Częstochowa, Armii Krajowej 13/15, 42-200 Częstochowa, Poland

**Keywords:** thioacids, phosphinothioic acids, selenoacids, phosphinoselenoic acids, chiral solvating agents, crystal structure, absolute configuration, conformation, X-ray crystallography

## Abstract

The aim of the research described in this publication is two-fold. The first is a detailed description of the synthesis of a series of compounds containing a stereogenic heteroatom, namely the optically active *P*-stereogenic derivatives of *tert*-butylarylphoshinic acids bearing sulfur or selenium. The second is a detailed discussion dedicated to the determination of their structures by an X-ray analysis. Such a determination is needed when considering optically active hetero-oxophosphoric acids as new chiral solvating agents, precursors of new chiral ionic liquids, or ligands in complexes serving as novel organometallic catalysts.

## 1. Introduction

Among chiral organophosphorus derivatives, phosphines are most extensively used in asymmetric synthesis (enantioselective or diastereoselective) as chiral inducers. Therefore, studies on their synthesis and characterization have significantly increased in recent years [1,2,3,4,5,6,7,8,9,10]. However, in a sharp contrast to this, *P*-stereogenic derivatives of phosphinic acid, which contain selenium or sulfur atoms, are, to date, much less investigated, even if they can be considered readily available, useful catalysts, and/or stoichiometric chiral auxiliaries. On the other hand, in recent decades, the determination of the enantiomeric excess of chiral compounds has been a very important research topic [11,12], mainly because of recent progress in the stereoselective (enantioselective) synthesis which created a need for an accurate and rapid determination of this parameter [13,14,15]. From the techniques used for this determination, NMR methods have so far found the widest application [11,12]. The simple but most advantageous experiments have been based on the use of chiral solvating agents (CSAs) [11,12,16]. Among the CSAs reported in the literature, (−)-(*S*)- and (+)-(*R*)-*tert*-butylphenylphosphinothioic acid (**1c**), which exist in only one tautomeric form and adopt one conformation in CCl_4_ solution [17], became widely applied in the NMR analysis of various chiral organic compounds [18,19]. We have also developed a new protocol for the synthesis of optically active, enantiomerically pure, dextrorotatory (*R*)-*tert*-butylphenylphosphinothioic acid **1c.** Now, it can be obtained in a reaction of racemic secondary *tert*-butylphenylphosphine oxide with elemental sulfur in the presence of an equimolar amount of the enantiomerically pure levorotatory enantiomer of (*S*)-α-phenylethylamine. It is obvious that with the use of the dextrorotatory enantiomer of α-phenylethylamine, the levorotatory enantiomer of this thioacid can be isolated [20,21].

**Scheme 1:**
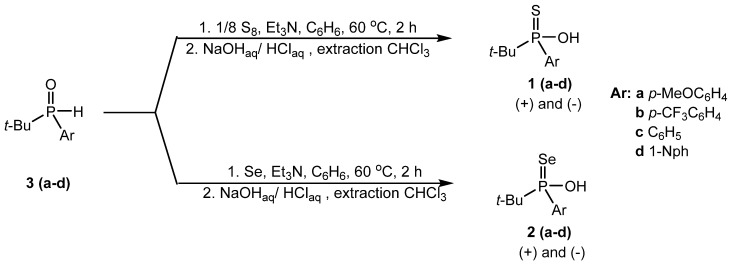
Synthesis of phosphinothioic acids (**1**) and phosphinoselenoic acids (**2**).

The crystal structure investigations of the enantiomeric forms of thioacids (**1**) and selenoacids (**2**) (Scheme 1), by using the X-ray method, were performed to obtain the critical and important information connected with their future application as ligands in organometallic catalysts. Thus, for example, after complexation with the copper-containing compounds, those chiral complexes could be used as the catalysts in the reactions in which copper acetate is routinely used as a catalyst (a “standard” asymmetric Henry reaction [22,23,24,25,26], the modified Ullmann [27,28], or Sonogashira [29] coupling reactions) and the reactions of oxidative polymerization [30]. Therefore, the newly proposed complexes of our enantiomerically pure chiral thio- and seleno-acids with metals could eliminate the use of extra chiral ligands in chemical reactions.

## 2. Results

### 2.1. Crystal and Molecular Structures of tert-Butyl-(4-methoxyphenyl)phosphinothioic Acid (***1a***) and tert-Butyl-(4-Trifluoromethylphenyl) Phosphinothioic Acid (***1b***)

**Figure 1:**
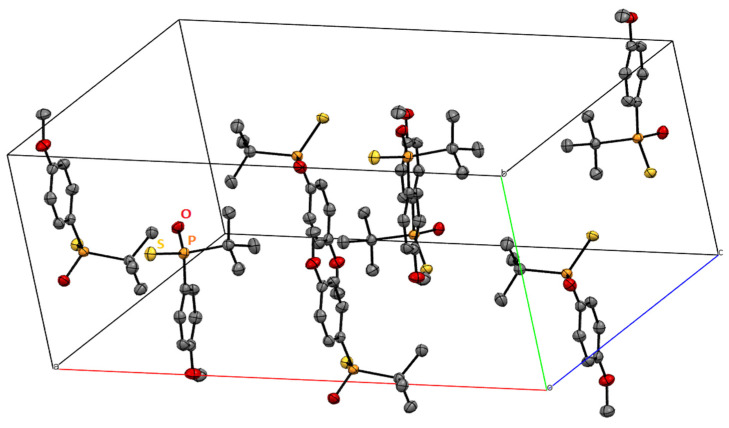
The unit-cell assembly of (*S*p)-*tert*-butyl-(4-methoxyphenyl) phosphinothioic acid **1a**. An asymmetric unit consists of two compound molecules. The thermal ellipsoids are drawn with 50% probability.

**Figure 2:**
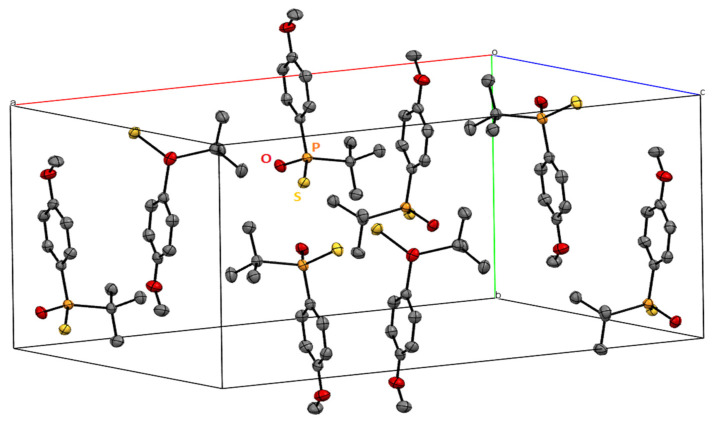
The unit-cell assembly of (*R*p)-*tert*-butyl-(4-methoxyphenyl)phosphinothioic acid **1a**. An asymmetric unit consists of two compound molecules. The displacement ellipsoids are drawn with 50% probability.

**Figure 3:**
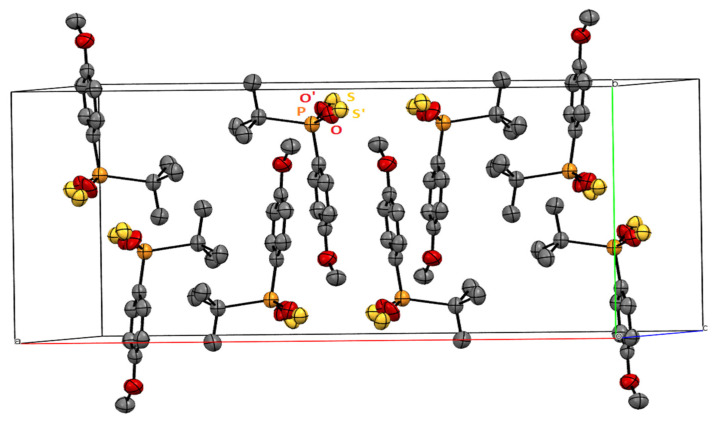
The unit-cell assembly of (*rac*)-*tert*-butyl-(4-methoxyphenyl)phosphinothioic acid **1a**. There is a single molecule of the compound in an asymmetric unit. The two positions of O and S atoms have been found in two independent positions, and their occupancies are 0.75 and 0.25. The ellipsoids are drawn with 50% probability.

**Figure 4:**
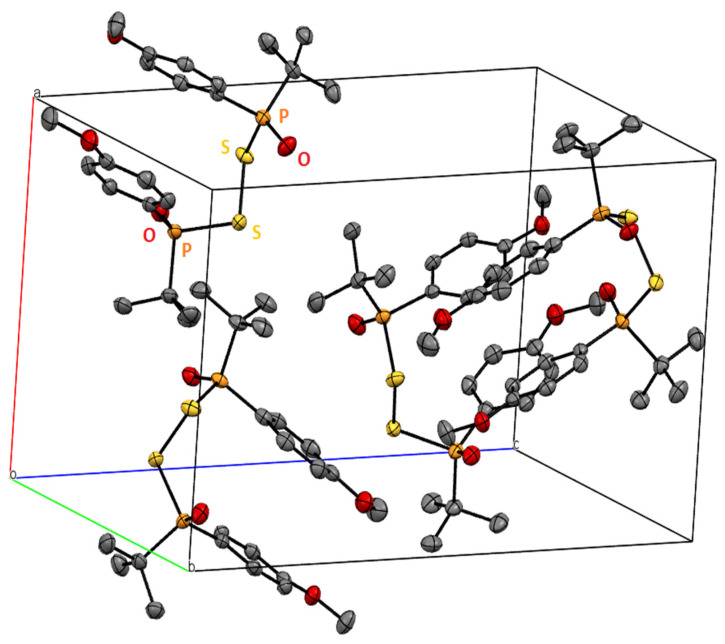
The unit-cell assembly of bis[(*S*p)-*tert*-butyl-(4-methoxyphenyl)phosphinothioic acid **1a** disulfide]. The entire molecule of disulfide has been found in the asymmetric unit. The displacement ellipsoids are drawn with 50% probability.

Both enantiomers of *tert*-butyl-(4-methoxyphenyl)phosphinothioic acid, (*S*p)-**1a** (CCDC accession code 1509139; Figure 1) and (*R*p)-**1a** (CCDC accession code 1589363; Figure 2), were crystallized in the monoclinic system, in space group C2, with the presence of two independent compound monomers in the respective asymmetric units. Racemic *tert*-butyl-(4-methoxyphenyl)phosphinothioic acid, (*rac*)-**1a** (Figure 3), is monoclinic and crystallizes in space group C2/c, with the presence of a single monomer in the asymmetric unit. This monomer shows disorder, i.e., the presence of two different positions of the O and S atoms (see Figure 3). The occupancies of the two disordered O and S atoms were 0.75 and 0.25 (also see “Figure 2” in [31]). The disulfide form of *tert-*butyl-(4-methoxyphenyl)phosphinothioic acid, bis-(*S*p)-**1a** (CCDC accession code 1589336; Figure 4), was crystallized in the orthorhombic system, and the space group was P212121 (Table S1—see Supplementary Materials). The entire disulfide has been found in the asymmetric unit (see Figure 4).

**Figure 5:**
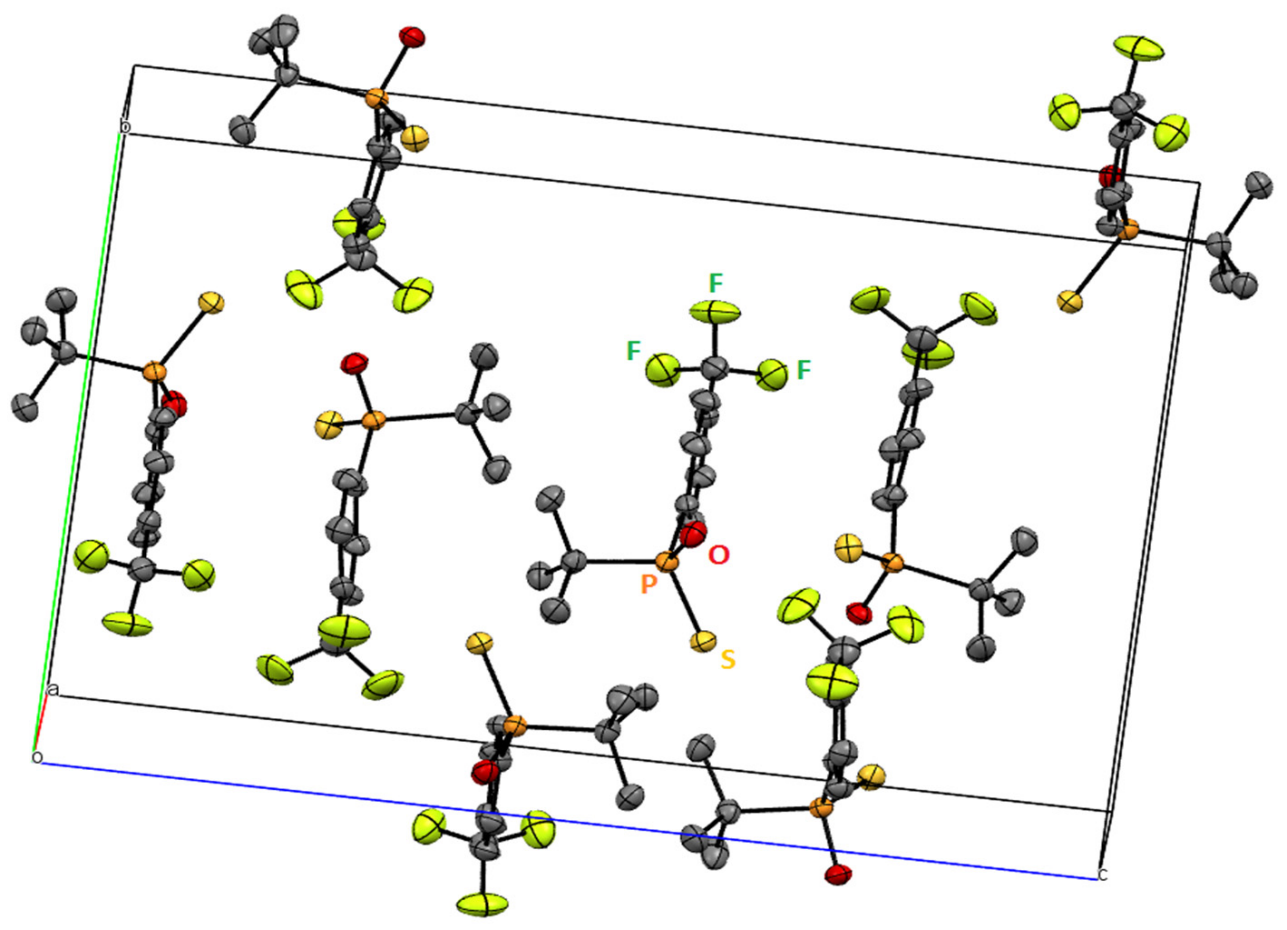
The unit cell of *tert*-butyl-(4-trifluoromethylphenyl)phosphinothioic acid (*R*p)-**1b**. The two independent compound molecules are present in an asymmetric unit. An asymmetric unit consists of two compound molecules. The displacement ellipsoids are drawn with 50% probability.

**Figure 6:**
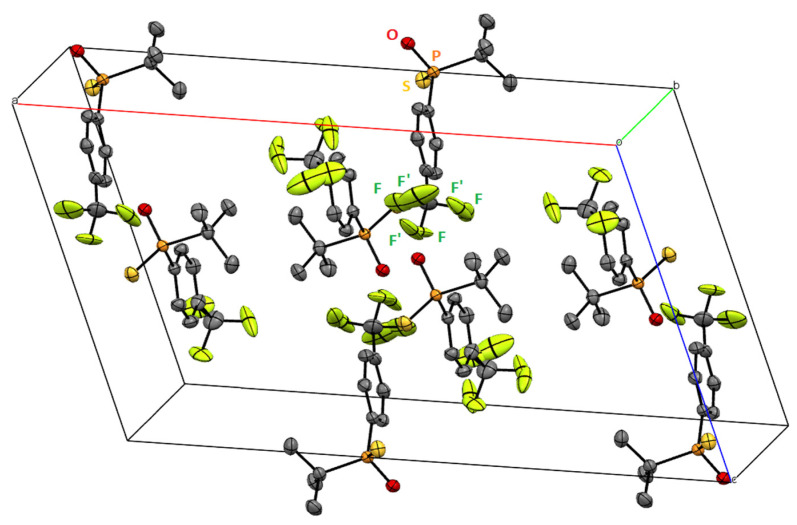
The unit cell of *tert*-butyl-(4-trifluoromethylphenyl)phosphinothioic acid, *rac*-**1b**. The single compound molecule has been found in the asymmetric unit. The CF_3_ group has been found in two independent positions, each with the occupancy of 50%. The disorder positions rotate by circa 20 degrees around the C-C bond. The thermal ellipsoids are drawn with 50% probability.

The enantiomer *R*p of *tert*-butyl-(4-trifluoromethylphenyl)phosphinothioic acid **1b**, (*R*p)-**1b** (CCDC accession code 2123210; Figure 5), was crystallized in the space group P2_1_2_1_2_1_ of the orthorhombic system, with two independent monomers present in the asymmetric unit. Racemic *tert*-butyl-(4-trifluoromethylphenyl)phosphinothioic acid **1b**, (*rac*)-**1b** (CCDC accession code 2123234; Figure 6), is monoclinic, and the space group is C2/c (Table S1—Supplementary Materials). The single monomer, which has been found in the asymmetric unit, shows two disorder positions of the CF3 group, each having the occupancy of 50%. The disorder positions show rotation by about 20 degrees around the C-C bond (see “Figure 4” in [31]).

These two determined structures of *tert*-butyl-(4-trifluoromethylphenyl)phosphinothioic acid **1b**, (*R*p)-**1b** and (*rac*)-**1b**, are not similar (not isostructural) to the respective structures of *tert*-butyl-(4-methoxyphenyl)phosphinothioic acid **1a**. The asymmetric unit of *tert*-butyl-(4-trifluoromethylphenyl)phosphinothioic acid (*R*p)-**1b** consists of two independent molecules, both with the absolute configuration, *R,* at the phosphorus atoms. Only, in this case, these two monomers formed a homodimer which lies entirely in an asymmetric unit (see “Figure 4” in [31]), whereas, for *tert*-butyl-(4-methoxyphenyl)phosphinothioic acid (*R*p)-**1a**, the dimer has to be built by applying the respective symmetry operations to each of the two molecules from the asymmetric unit (see “Figures 2 and 3” in [31]). For *tert*-butyl(4-trifluoromethylphenyl)phosphinothioic acid (*R*p)-**1b**, only such homodimer as that seen in “Figure 4” in [31] serves as a building block for the entire crystal lattice.

All three forms of *tert*-butyl-(4-methoxyphenyl)phosphinothioic acid **1a**, (*S*p)-**1a**, (*R*p)-**1a**, and (*rac*)-(**1a**), and *tert*-butyl-(4-trifluoromethylphenyl)phosphinothioic acid (*R*p)-**1b**, form homodimers (i.e., *S*p/*S*p or *R*p/*R*p) in their crystal lattices. The stability of these four homodimers is supported by the stacking of the planar six-membered phenyl rings. For the details of the homodimer assembly of phosphinothioic acids **1a** and **1b**, see “Figures 3 and 4” in [31].

The crystal lattice of racemic *tert*-butyl-(4-trifluoromethylphenyl) phosphinothioic acid **1b** reveals the presence of heterodimers (*R*p/*S*p). For details, see “Figure 4” in [31]. The molecules of **1b** in racemic crystal do not dimerize by stacking of the planar six-membered carbon rings. The deviation from planarity (an “imperfect stacking”) of these rings, which is about 20 degrees, and the glide between these rings, are both most likely due to the steric hindrance of the large substituents attached to those rings (see “Figure 4” in [31]). As mentioned above and shown in “Figure 4” in [31], the two molecules which create the heterodimer differ in the absolute configuration at the phosphorus atoms. The opposite configurations at the P atoms in both dimer components is an obvious result of the application of the center-of-symmetry operation (−x, −y, −z) to each of the molecules from the (hetero)dimer.

### 2.2. Crystal and Molecular Structures of tert-Butyl-(4-methoxyphenyl)phosphinoselenoic Acid (Sp)-***2a***, (Rp)-***2a***, and (rac)-***2a***

**Figure 7:**
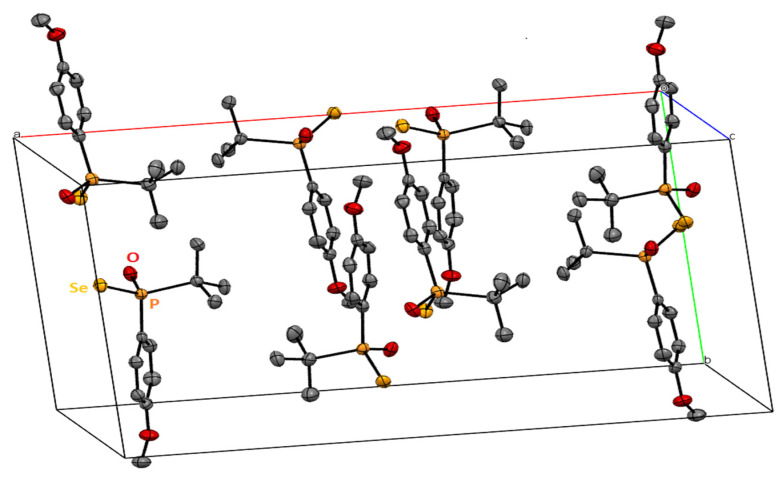
The unit-cell assembly of *tert*-butyl-(4-methoxyphenyl)phosphinoselenoic acid (*S*p)-**2a**. An asymmetric unit consists of two compound molecules. The ellipsoids are drawn with 50% probability.

**Figure 8:**
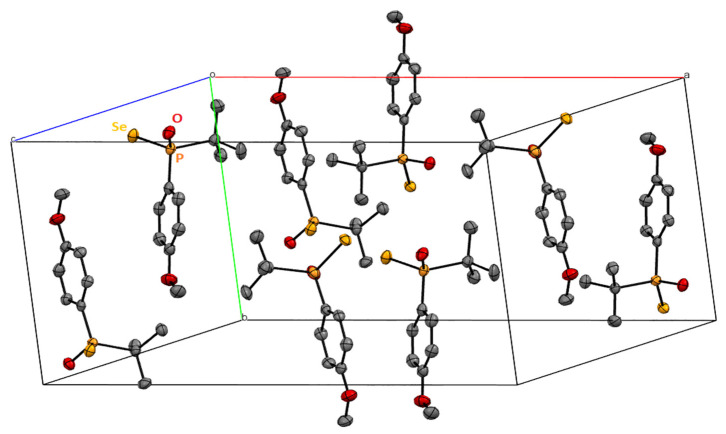
The unit-cell assembly of *tert*-butyl-(4-methoxyphenyl)phosphinoselenoic acid (*R*p)-**2a**. An asymmetric unit consists of two compound molecules. The displacement ellipsoids are drawn with 50% probability.

**Figure 9:**
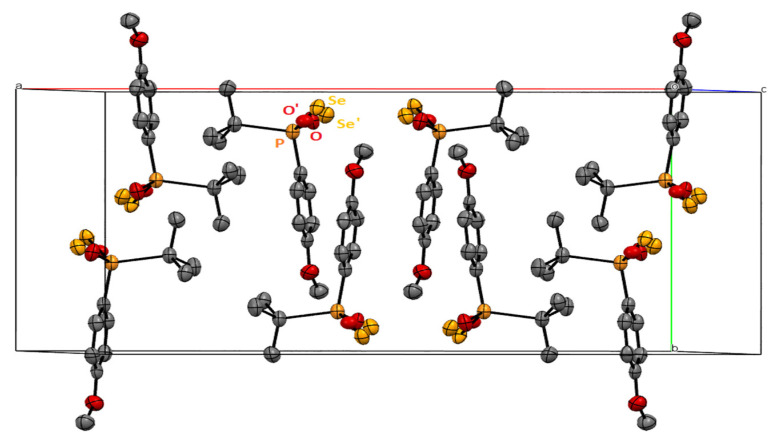
The unit-cell assembly of *tert*-butyl-(4-methoxyphenyl)phosphinoselenoic acid (*rac*)-**2a**. There is a single molecule of the compound in an asymmetric unit. The two positions of O and Se atoms have been found in two independent positions, and their occupancies are 0.89 and 0.11. The thermal ellipsoids are drawn with 50% probability.

Both enantiomers *S*p and *R*p of *tert-*butyl-(4-methoxyphenyl)phosphinoselenoic acid **2a**, (*S*p)-**2a** (CCDC accession code 2123219; Figure 7) and (*R*p)-**2a** (CCDC accession code 1509140; Figure 8), were crystallized in space group C2 of the monoclinic system. The two monomers are present in the asymmetric units of both compounds (see Table S2). The racemic *tert*-butyl-(4-methoxyphenyl)phosphinoselenoic acid, (*rac*)-**2a** (CCDC accession code 2123223), is monoclinic, and the space group is C2/c. The asymmetric unit contains a single monomer which contains two positionally disordered O and Se atoms (Figure 9).

The asymmetric unit of *tert*-butyl-(4-methoxyphenyl)phosphinoselenoic acid (*S*p)-**2a** contains two independent molecules, both having an absolute configuration, *S,* at the phosphorus atoms (Figure 7). In the asymmetric unit of (*R*p)-**2a**, similarly, there are two independent molecules, with the absolute configuration, *R,* at both phosphorus atoms (Figure 8). The racemic **2a** contains a single monomer in the asymmetric unit (Figure 9). This molecule is disordered in a similar manner as we found in the structure of the sulfur-containing counterpart, presented in Figure 3. The disorder components in the (*rac*)-**2a** monomer differ (in comparison with (*rac*)-**1a**) in the occupancy ratio of the disordered Se and O atoms, which is equal to 0.89/0.11. For details, see “Figure 1” in [31].

We found that all three *tert*-butyl-(4-methoxyphenyl)phosphinoselenoic acids, (*S*p)-**2a**, (*R*p)-**2a**, and (*rac*)-**2a** (see Figure 7, Figure 8 and Figure 9), are isostructural with the respective sulfur-containing counterparts (see Figure 1, Figure 2 and Figure 3). The comparison of the unit-cell dimensions (*a*, *b*, *c*, and volume) in Tables S1 and S2 showed the longer lengths of the unit-cell dimensions (*a*, *b*, *c*) and the larger values of the unit-cell volumes for phosphinoselenoic acids than in the respective sulfur-containing compounds. Obviously, the consequent increase of the unit-cell parameters and volumes was due to the presence of a larger atom, i.e., selenium in comparison to sulfur.

All three *tert*-butyl-(4-methoxyphenyl)phosphinoselenoic acids: (*S*p)-**2a**, (*R*p)-**2a**, and (*rac*)-**2a**, formed homodimers in their crystal lattices in a similar manner to the three respective *tert*-butyl-paramethoxyphenyl phosphinothioic acids: (*S*p)-**1a**, (*R*p)-**1a**, and (*rac*)-**1a.** For details, compare “Figure 2” in [31] with “Figure 3” in [31]. In particular, the stability of the homodimers of all three *tert-*butyl-(4-methoxyphenyl)phosphinoselenoic acids: (*S*p)-**2a**, (*R*p)-**2a**, and (*rac*)-**2a**, was supported by the stacking of the planar six-membered rings. Similarly, the phosphinoselenoic acid dimers must be created by applying the particular symmetry operations to each of the two monomers from the respective asymmetric units. Only these homodimers constitute the building blocks for the entire crystal lattices.

## 3. Experimental

### 3.1. Synthesis

#### 3.1.1. General Information

The NMR spectra were recorded on Bruker Avance AV 200 or Bruker AV Neo 400 Spectrometers (^1^H, ^13^C, and ^31^P) in CDCl_3_, (CD_3_)_2_C(O), or C_6_D_6_ (Billerica, MA, USA). IR spectra were recorded as thin films on Si p-type plates by the using Jasco Joel FT/IR 6200 Fourier Transform Infrared Spectrometer, 400–4000 cm^−1^ (Tokyo, Japan). UV spectra were recorded in MeOH by using the UV-VIS Shimadzu 2600 Spectrophotometer (Kyoto, Japan).

Mass spectral data were collected on the MAT95-Finnigan Spectrometer (Finnigan MAT, Bremen, Germany). Optical rotation was determined on the 241 MC-Perkin Elmer polarimeter (Perkin Elmer, Vienna, Austria) at room temperature. Melting points were determined on Betius apparatus (PHMKVEB Analytik, Dresden, Germany) and were uncorrected. Column chromatography was performed on Merk Silica Gel (F254 60, 270–400 mesh). Merck Silica F254 plates (Rahway, NJ, USA) were used for thin-layer chromatography and visualization was affected with UV light (254 nm). Elemental analyses were performed in the Microanalytical Laboratory of the Centre of Molecular and Macromolecular Studies, PAS. Reactions were performed under a blanket of dry nitrogen.

#### 3.1.2. General Procedure and Characterization of the Racemic *tert*-Butylarylphosphinothioic Acids (**1a**–**d**)

A mixture of racemic *tert*-butylarylphosphine oxide **3**, triethylamine (1 eq), and sulfur (1 eq) in anhydrous benzene was heated at 80 °C under nitrogen for 2 h. The reaction mixture was cooled to room temperature, aqueous sodium hydroxide solution (10%) was added, and the organic layer was separated. The aqueous layer was extracted with chloroform and acidified with hydrochloric acid (10%) to pH 1, and then extracted with chloroform. The combined chloroform extracts were dried over magnesium sulfate, filtered, and concentrated to yield crude products as crystals. Yields and physicochemical and spectroscopic data of the obtained products are summarized in the Supplementary Materials. The Supplementary Materials also contain copies of the original NMR (^1^H, ^13^C, and ^31^P), IR, and UV-vis spectra.

#### 3.1.3. General Procedure and Characterization of the Racemic *tert*-Butylarylphosphinoselenoic Acids (**2a**–**d**)

A mixture of racemic *tert*-butylarylphosphine oxide **3**, triethylamine (1 eq), and selenium (1 eq) in anhydrous benzene was heated at 60 °C under nitrogen for 2 h. The reaction mixture was cooled to room temperature, aqueous sodium hydroxide solution (10%) was added, and the organic layer was separated. The aqueous layer was extracted with chloroform and acidified with hydrochloric acid (10%) to pH 1, and then extracted with chloroform. The combined chloroform extracts were dried over magnesium sulfate, filtered, and concentrated to yield crude products as crystals. Yields and physicochemical and spectroscopic data of the obtained products are summarized in the Supplementary Materials. The Supplementary Materials also contain copies of the original NMR (^1^H, ^31^P), IR, and UV-vis spectra.

#### 3.1.4. Synthetic Procedure and Characterization of Racemic *tert*-Butylarylphosphine Oxides (**3a**–**d**)

To the solution of dichlorophosphine in dry diethyl ether, a solution of alkyl- or aryl-magnesium bromide in dry THF, at −30 °C under argon atmosphere, was slowly added. The mixture was stirred at this temperature for 2 h and then the cooling bath was removed. The reaction was performed at room temperature for the next 12 h. After 2 h of refluxing, the solution was cooled to 5 °C and 6 M aqueous HCI was added. The product was then extracted with chloroform. The organic layer was washed with 0.7 M NaOH and water, dried over MgSO_4_, and concentrated at reduced pressure. Yields and physicochemical and spectroscopic data of the obtained products are summarized in the Supplementary Materials.


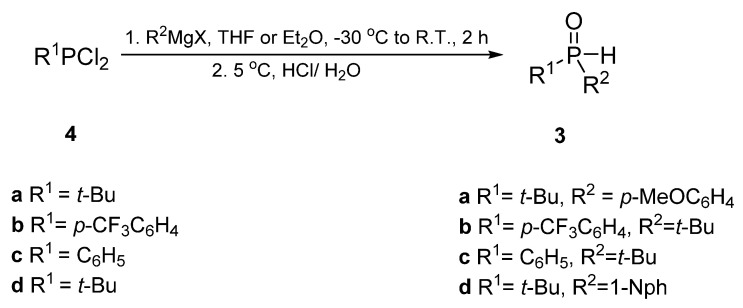


#### 3.1.5. X-ray Crystallography

Here, we provide the information about the X-ray structures of phosphinothioic (**1**) and phosphinoselenoic (**2**) acids (Scheme 1). Phosphinothioic acids **1a** and **1b** and phosphinoselenoic acids **2a** and **2b** were synthesized according to our earlier described procedure [32,33]. It should be noted here that the X-ray structure of the levorotatory (*S*) enantiomer of *tert*-butylphenylphosphinoselenoic acid (**2c**) has been reported [34].

The list of the X-ray structures of phosphinothioic acids contains all three forms of *tert*-butyl-(4-methoxyphenyl)phosphinothioic acid **1a**: *R*p-**1a**, *S*p-**1a**, and *rac-***1a**, and two forms of *tert*-butyl-(4-trifluoromethylphenyl)phosphinothioic acid **1b**: *R*p-**1b** and *rac-***1b**. Unexpectedly, we obtained the structure of *tert*-butyl-(4-methoxyphenyl)phosphinothioic acid (*S*p)-**1a** in a disulfide form. For crystal data and refinement details, see Table S1 in the Supplementary Materials.

The list of the determined X-ray structures of phosphinoselenoic acids contains all three forms (*R*p, *S*p, and *rac*) of *tert-*butyl-(4-methoxyphenyl)phosphinoselenoic acid **2a** (see Table S2 in the Supplementary Materials).

The diffraction data for all structures determined in this work (see Tables S1 and S2) have been collected with an XtaLAB Synergy Dualflex Pilatus 300 K (Rigaku, Tokyo, Japan) diffractometer using the PhotonJet microfocus X-ray Source. The data were collected at a temperature of 100 K using CuKα radiation (λ = 1.54184Å) and the ω-scan technique. Absorption corrections which we performed using the CrysAlis PRO program (RigakuV1.171.41.89A, 2020) [35] were based on the indexing of the crystal faces. All structures were solved by direct methods of the SHELXT-2018/2 program and followed by the Fourier and difference Fourier syntheses. We refined all the structures with the SHELXL-2018/3 software [36] using the full-matrix least-squares on F^2^. The anisotropic displacement parameters were refined for all non-hydrogen atoms. The hydrogen atoms were placed in idealized positions and refined with isotropic displacement parameters in a riding manner. The Mercury program has been used for molecular graphics [37].

The crystallographic refinements of all structures yielded very good values of *R1*-factors and unequivocal values for the Flack *x* parameters (in all structures where the determination of the absolute configuration was applicable [31]). The crystal data and experimental details are provided in Tables S1 and S2 (Supplementary Materials).

## 4. Conclusions

We have synthesized a series of optically active *P*-stereogenic derivatives of *tert*-butylarylphosphinic acids bearing sulfur or selenium. They have been subjected to an X-ray analysis which allowed to precisely determine their structures. Such a determination is vital from the point of view of their application as potential new chiral solvating agents, precursors of new chiral ionic liquids, or ligands in novel organometallic catalysts.

The asymmetric units of the determined compounds contained either single-compound molecules or two independent molecules which differ in their conformation. Additionally, the structure of an unexpected disulfide form of *S*p-**1a** has been obtained, with the presence of an entire disulfide molecule in an asymmetric unit. The racemic forms showed the molecular disorder at the particular atoms or groups. In most cases, the molecules formed homodimers (*R*p/*R*p or *S*p/*S*p) in their crystal lattices; however, interestingly, the crystal lattice of racemic **1b** uniquely showed the presence of heterodimers (*R*p/*S*p).

## 5. CCDC Accession Codes

CCDC depositions 1509139, 1589363, 1589364, 1589336, 2123210, and 2123234 contain the supplementary crystallographic data for *tert-*butyl-(4-methoxyphenyl)phosphinothioic acids (*S*p)-**1a**, (*R*p)-**1a**, (*rac*)-**1a**, and bis-[(*S*p)-**1a**] disulfide, and *tert*-butyl-(4-trifluoromethylphenyl) phosphinothioic acids (*R*p)-**1b** and (*rac)*-**1b**, respectively. CCDC depositions 2123219, 1509140, and 2123223 contain the supplementary crystallographic data for *tert*-butyl-(4-methoxyphenyl) phosphinoselenoic acids (*S*p)-**2a**, (*R*p)-**2a**, and (*rac*)-**2a**, respectively.

## Data Availability

These data can be obtained free of charge via https://www.ccdc.cam.ac.uk/structures/ (accessed on 1 November 2020), or by e-mailing data_request@ccdc.cam.ac.uk, or by contacting The Cambridge Crystallographic Data Centre, 12 Union Road, Cambridge CB2 1EZ, UK; fax: +44(0)1223-336033.

## Supplementary Materials

The following supporting information can be downloaded at: https://www.mdpi.com/article/10.3390/molecules28114298/s1, Table S1. Crystal data and experimental details of determined phosphinothioic acids **1a**–**1b**. Table S2. Crystal data and experimental details of determined phosphinoselenoic acid **2a**.
