# Supplementary material for: New Optically Active tert-Butylarylthiophosphinic Acids and Their Selenium Analogues as the Potential Synthons of Supramolecular Organometallic Complexes: Syntheses and Crystallographic Structure Determination
Source: Molecules. 2023 May 24;28(11):4298. doi: 10.3390/molecules28114298 (PMC10254544; doi:10.3390/molecules28114298)
Supplement: Supplementary file 1 [file molecules-28-04298-s001.zip › molecules-2388832-supplementary.pdf]

Supporting Information for:

New optically active *tert*-butylarylthiophosphinic acids and their selenium analogues as the potential synthons of supramolecular organometallic complexes: syntheses and crystallographic structure determination

Jarosław Błaszczyk[1,*], Bogdan Bujnicki[1], Patrycja Pokora-Sobczak[1], Grażyna Mielniczak[1], Lesław Sieroń[2], Piotr Kiełbasiński[1] and Józef Drabowicz[1,3,*]

[1]Division of Organic Chemistry, Centre of Molecular and Macromolecular Studies, Polish Academy of Sciences, Sienkiewicza 112, 90-363 Łódź, Poland; bogdan.bujnicki@cbmm.lodz.pl (B.B.);

patrycja.pokora-sobczak@cbmm.lodz.pl (P.P.-S.); grazyna.mielniczak@cbmm.lodz.pl (G.M.); piotr.kielbasinski@cbmm.lodz.pl (P.K.)

[2]Institute of General and Ecological Chemistry, Lodz University of Technology, Żeromskiego 116, 90-924 Łódź, Poland; leslaw.sieron@p.lodz.pl

[3]Institute of Chemistry, Jan Długosz University in Częstochowa, Armii Krajowej 13/15, 42-200 Częstochowa, Poland

**Correspondence: jaroslaw.blaszczyk@cbmm.lodz.pl (J.B.); jozef.drabowicz@cbmm.lodz.pl (J.D.)*

Table of Contents:

S1. Experimental Procedures 2-5
S2. NMR-Spectra 6-22
S3. UV-VIS Spectra 23-25
S4. IR Spectra 26-28
S5. Crystal data and experimental details 29-31

S1. Experimental Procedures

General procedure and characterization of the racemic tert-butylarylphosphinothioic acids (1a-d)

A mixture of racemic *tert*-butylarylphosphine oxide **3**, triethylamine (1eq), and sulfur (1eq) in anhydrous benzene was heated at 80 °C under nitrogen for 2 h. The reaction mixture was cooled to room temperature, aqueous sodium hydroxide solution (10%) was added and the organic layer was separated. The aqueous layer was extracted with chloroform and acidified with hydrochloric acid (10%) to pH 1 and extracted with chloroform. The combined chloroform extracts were dried over magnesium sulfate, filtered, and concentrated to give crude products as crystals.

tert-butyl-(4-methoxyphenyl)phosphinothioic acid (**1a**)

Yield: 60%, (4.3 mmol, 1.049g), white solid
m.p. : 126-128 °C
^{1}H NMR (200 MHz, $CDCl_3$) δ (ppm): 7.73 – 7.54 (m, 2H), 6.81 (dd, *J* = 8.9, 2.6 Hz, 2H), 3.83 (s, 3H), 1.12 (d, *J* = 17.6 Hz, 9H)
^{31}P NMR (81 MHz, $CDCl_3$) δ (ppm): 97.98
^{13}C NMR (101 MHz, $CDCl_3$) δ (ppm): 162.00 (d, *J* = 3.2 Hz), 134.12 (d, *J* = 11.5 Hz), 122.81 (d, *J* = 100.6 Hz), 113.06 (d, *J* = 13.4 Hz), 55.13, 36.23 (d, *J* = 74.7 Hz), 24.13
IR (KBr): ν =2967s; 1595s; 1500s; 1457w; 1363w; 1294m; 1258s; 1180w; 1112s; 1019w; 907m; 812w; 655w; 634w; 621m; 586w; 422w cm^{-1}.
UV (MeOH): 233, 274, 282 nm
MS (CI): m/z = 245 [M+1]
Anal. Calcd. for $C_{11}H_{17}O_2PS$: C, 54.08; H, 7.01; S,13.12. Found: C,54.01; H, 6.89; S, 12.98

tert-butyl-(4-trifluoromethylphenyl)phosphinothioic acid (**1b**)

Yield: 67%, (3.0 mmol, 0.846g), white solid
m.p. :110-112 °C
^{1}H NMR (500 MHz, $CDCl_3$) δ (ppm): 7.92 (td, *J* = 11.6, 8.0 Hz, 2H), 7.65 (td, *J* = 8.8, 2.5 Hz, 2H), 1.12 (dd, *J* = 17.9, 2.9 Hz, 9H)
^{31}P NMR (202 MHz, C_6D_6) δ (ppm): 97.06 (d, *J* = 7.2 Hz) C_6D_6
^{13}C NMR (101 MHz, $CDCl_3$) δ (ppm): 136.15 (d, *J* = 91.2 Hz), 133.29 (dd, *J* = 32.7, 3.2 Hz), 132.70 (d, *J* = 10.5 Hz), 124.52 (dq, *J* = 12.0, 3.9 Hz), 123.50 (q, *J*= 272.6), 36.32 (d, *J* = 73.1 Hz), 24.0
^{19}F NMR (376 MHz, $CDCl_3$) δ (ppm): -62.52
IR (KBr): ν =2969m; 1540w; 1477w; 1396w; 1325s; 1171m; 1132m; 1063s; 1018w; 921w; 883w; 712m; 676m; 601w; 507w cm^{-1}.
UV (MeOH): 234, 272, 280 nm
MS (CI): m/z = 283.1 [M+1]

tert-butylphenylphosphinothioic acid (**1c**)

Yield: 96%, (5.3 mmol,1.130g), white solid
m.p. : 103-106 °C
^{1}H NMR (400 MHz, $CDCl_3$) δ (ppm): 7.82- 7.74 (m, 2H), 7.48- 7.41 (m, 1H), 7.37 (tdd, *J* = 6.5, 3.3, 1.4 Hz, 2H), 1.09 (d, *J* = 17.7 Hz, 1H)

^{31}P NMR (162 MHz, $CDCl_3$) δ (ppm): 97.38
^{13}C NMR (101 MHz, $CDCl_3$) δ (ppm): 132.31 (d, *J* = 10.2 Hz), 131.72 (d, *J* = 94.7 Hz), 131.53 (d, *J* = 3.1 Hz), 127.72 (d, *J* = 12.3 Hz), 36.23 (d, *J* = 72.9 Hz), 24.08 (d, *J* = 1.7 Hz)
MS (CI): m/z = 215.1 [M+1]

tert-butyl-1-naphtylphosphinothioic acid (**1d**)

Crystallization from a hexane: CH_2Cl_2 mixture (5:1) gave pure thioacid **1d** as white crystals
Yield: 98%, (6.3 mmol, 1.673 g)
m.p. : 188-190 °C
^{1}H NMR (200 MHz, $CDCl_3$) δ (ppm): 9.10-9.05 (m, 1H), 8.22-8.11 (m, 1H), 7.89 -7. 78 (m, 2H), 7.51 -7.46 (m, 2H), 7.39-7.30 (m, 1H), 1.16 (d, *J*=17.86 Hz, 9H)
^{31}P NMR (81 MHz, $CDCl_3$) δ (ppm): 99.4
^{13}C NMR (101 MHz, $CDCl_3$) δ (ppm): 135.06 (d, *J* = 9.4 Hz), 133.49 (dd, *J* = 38.2, 10.5 Hz), 133.15 (d, *J* = 3.2 Hz), 128.66, 127.79 (d, *J* = 2.8 Hz), 127.30 (d, *J* = 87.0 Hz), 126.41, 125.82, 123.83 (d, *J* = 13.7 Hz), 38.01 (d, *J* = 72.3 Hz), 24.76 (d, *J* = 2.2 Hz)
IR (KBr): ν =2856s; 1590w; 1508m; 1475m; 1458m; 1392w; 1363w; 1207w; 981w; 914s; 803s; 772s; 684s; 665s; 591m; 462m; 436m cm^{-1}
UV (MeOH): 288 nm
MS (EI): m/z = 264.1 [M+1]
Anal. Calcd. for $C_{14}H_{17}OPS$: C, 63.62; H, 6.48, S:12,13. Found: C,63,78; H, 6.43; S: 12,27

General procedure and characterization of the racemic tert-butylarylphosphino-selenoic acids (2a-d)

A mixture of racemic *tert*-butylarylphosphine oxide **3**, triethylamine (1eq), and selenium (1eq) in anhydrous benzene was heated at 60 °C under nitrogen for 2 h. The reaction mixture was cooled to room temperature, aqueous sodium hydroxide solution (10%) was added and the organic layer was separated. The aqueous layer was extracted with chloroform and acidified with hydrochloric acid (10%) to pH 1 and extracted with chloroform. The combined chloroform extracts were dried over magnesium sulfate, filtered, and concentrated to give crude products as crystals.

tert-butyl-(4-methoxyphenyl)phosphinoselenoic acid (**2a**)

Yield: 67%, (3.2mmol, 0,931g), white solid
m.p. : 120-123 °C
^{1}H NMR (400 MHz, $CDCl_3$) δ (ppm): 7.71 (dd, *J* = 11.4, 8.4 Hz, 2H), 7.71 (dd, *J* = 11.4, 8.4 Hz, 1H), 6.55 (s, 1H), 3.83 (s, 1H), 1.10 (d, *J* = 18.1 Hz, 9H)
^{31}P NMR (162 MHz, $CDCl_3$) δ (ppm): 96.39
^{13}C NMR (101 MHz, $CDCl_3$) δ (ppm): 162.25 (d, *J* = 3.1 Hz), 134.23 (d, *J* = 11.9 Hz), 123.16 (d, *J* = 89.3 Hz), 113.17 (d, *J* = 13.4 Hz), 55.29, 37.15 (d, *J* = 63.3 Hz), 24.31 (d, *J* = 2.4 Hz)
IR (KBr): ν =2971s; 1594s; 1502m; 1362w; 1294m; 1260s; 1181w; 1112s; 1019m; 919s; 849w; 680s; 652w; 526w; 422w cm^{-1}.
UV (MeOH): 262, 268, 275 nm
MS (GC): m/z = 292.0 [M+1]
Anal. Calcd. for $C_{11}H_{17}O_2PSe$: C, 45.37; H, 5.88. Found: C, 45.10; H, 5.99

tert-butyl-(4-trifluoromethylphenyl)phosphinoselenoic acid (**2b**)

Yield: 76%, (3.0mmol, 0.987g), white solid
m.p.: 128-129 °C
^{1}H NMR (400 MHz, $CDCl_3$) δ (ppm): 7.89 (dd, *J* = 11.4, 8.1 Hz, 2H), 7.59 (dd, *J* = 8.4, 2.7 Hz, 2H), 1.15 (d, *J* = 18.4 Hz, 9H).
^{31}P NMR (202 MHz, C_6D_6) δ (ppm): 96.73
^{13}C NMR (125 MHz, $CDCl_3$) δ (ppm): 162.25 (d, *J* = 3.1 Hz), 134.23 (d, *J* = 11.9 Hz), 123.16 (d, *J* = 89.3 Hz), 113.17 (d, *J* = 13.4 Hz), 55.29, 37.15 (d, *J* = 63.3 Hz), 24.31 (d, *J* = 2.4 Hz)
IR (KBr): ν =2927w; 1540w; 1475w; 1396w; 1323s; 1170m; 1132m; 1063m; 1017s; 913w; 883w; 706w; 635w; 565w cm^{-1}.
UV (MeOH): 269, 276 nm

tert-butylphenylphosphinoselenoic acid (**2c**)

Crystallization from a hexane: CH_2Cl_2 mixture (5:1) gave pure **2c** as white crystals:
Yield: 80%, (4.4 mmol, 1.158 g)
m.p. : 98-99 °C
^{1}H NMR (400 MHz, $CDCl_3$) δ (ppm): 7.86-7.77 (m, 2H), 7.49-7.42 (m, 1H), 7.39 (ddd, *J* = 7.0, 5.5, 2.6 Hz, 2H), 1.11 (d, *J* = 18.1 Hz, 9H)
^{31}P NMR (162 MHz, $CDCl_3$) δ (ppm): 98.26
^{13}C NMR (125 MHz, $CDCl_3$) δ (ppm): 132.69 (d, *J* = 82.6 Hz), 132.43 (d, *J* = 10.5 Hz), 131.81 (d, *J* = 3.2 Hz), 37.14 (d, *J* = 62.0 Hz), 24.39 (d, *J* = 2.5 Hz).

tert-butyl-1-naphthylphosphinoselenoic acid (**2d**)

Crystallization from a hexane: CH_2Cl_2 mixture (5:1) gave pure **2d** as white crystals
Yield: 98% (6.3 mmol, 1.673 g)
m.p. : 168-170 °C
^{1}H NMR (400 MHz, $(CD_3)_2C=O$) δ (ppm): 9.64-9.35 (m, 1H), 8.45-8.35 (m, 1H), 8.14 (dd, *J* = 8.2, 2.0 Hz, 1H), 8.00 (dt, *J* = 7.5, 2.1 Hz, 1H), 7.68-7.61 (m, 1H), 7.58 (ddt, *J* = 8.0, 6.8, 5.2 Hz, 2H), 1.20 (d, *J* = 17.7 Hz, 9H)
^{31}P NMR (162 MHz, $CDCl_3$) δ (ppm): 94.59
^{13}C NMR (101 MHz, $CDCl_3$) δ (ppm): 134.99, 133.80 (d, *J* = 9.8 Hz), 133.20 (d, *J* = 3.3 Hz), 133.10 (d, *J* = 10.6 Hz), 128.65, 127.74 (d, *J* = 3.2 Hz), 127.20 (d, *J* = 75.1 Hz), 126.32, 125.95, 123.82 (d, *J* = 13.9 Hz), 38.79 (d, *J* = 61.3 Hz), 24.93 (d, *J* = 2.8 Hz)
IR (KBr): ν =2965s; 1508w; 1476w; 1458w; 1363w; 1204w; 1145w; 979w; 903w; 803s; 772s; 638m; 584m; 547m; 507w; 453w; 435w cm^{-1}.
UV (MeOH): 288 nm
MS (CI): m/z = 313 [M+1]
Anal. Calcd. for $C_{14}H_{17}OPSe$: C, 54.03; H, 5.51. Found: C, 54,21; H, 5.79

Procedure and characterization of the racemic tert-butylarylphosphine oxides (3a-d)

To the solution of dichlorophosphine in dry diethyl ether a solution of alkyl or arylmagnesium bromide in dry THF, at -30 ^oC under argon atmosphere was slowly added. The mixture was stirred at this temperature for 2 hours and then the cooling bath was removed. The reaction was performed at room temperature for next 12 hours. After 2 h of refluxing the solution was

cooled to 5 °C and 6M aqueous HCl was dropped. The product was extracted with chloroform. The organic layer was washed with 0.7M NaOH and water, dried over $MgSO_4$ and concentrated at reduced pressure.

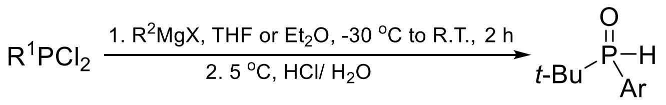

4
a R^1=*t*-Bu
b R^1=Ar= *p*-$CF_3C_6H_4$
c R^1=Ar= C_6H_5
d R^1=*t*-Bu

3
a R^1=*t*-Bu, R^2=Ar=*p*-$MeOC_6H_4$
b R^1=Ar= *p*-$CF_3C_6H_4$, R^2=*t*-Bu
c R^1=Ar= C_6H_5, R^2=*t*-Bu
d R^1=*t*-Bu, R^2=Ar=1-Nph

tert-butyl-(4-methoxyphenyl)phosphine oxide (**3a**)

Yield: 95%, (20.0 mmol, 4.03g), white solid
m.p.: 66-67 ºC
^{1}H NMR (200 MHz, $CDCl_3$) δ (ppm): 7.75 -7.41 (m, 2H), 7.08- 6.87 (m, 2H), 6.99 (d, *J*= 451.0, 1H), 3.84 (s, 3H), 1.11 (d, *J* = 16.6 Hz, 9H)
^{31}P NMR (81 MHz, $CDCl_3$) δ (ppm): 47.28
MS (CI): m/z = 213.1 [M+1]

tert-butyl-(4-trifluoromethylphenyl) phosphine oxide (**3b**)

Yield: 90%, (13.1 mmol, 3.28 g), white solid.
m.p.: 65-66 ºC
^{1}H NMR (200 MHz, $CDCl_3$) δ (ppm): 7.86 (d, *J*= 8.3 Hz, 1H), 7.82 – 7.71 (m, 3H), 7.12 (d, *J*= 458.0 Hz 1H), 1.17 (d, *J* = 17.2 Hz, 9H)
^{31}P NMR (81 MHz, $CDCl_3$) δ (ppm): 45.62
MS (TOF-ES+): m/z= 251.03 [M+1]

tert-butylphenylphosphine oxide (**3c**) was synthesized by a known method[19]

Yield: 95%, (18.0 mmol, 3.28 g), white solid
^{1}H NMR (200 MHz, $CDCl_3$) δ (ppm): 7.67 (ddt, *J* = 12.2, 6.9, 1.4 Hz, 2H), 7.59 (d, *J* = 453.1 Hz, 1H), 7.58 – 7.53 (m, 1H), 7.52 – 7.44 (m, 2H), 1.14 (d, *J* = 16.6 Hz, 9H)
^{31}P NMR (81 MHz, $CDCl_3$) δ (ppm): 47.62

tert-butyl-1-naphthylphosphine oxide (**3d**) was synthesized by a known method[28]

Yield: 75%, (94.0 mmol, 21.8g), white solid
m.p.: 130-132 °C
^{1}H NMR (200 MHz, $CDCl_3$) δ (ppm): 8.73-8.68 (m, 1H), 8.06-8.02(d, *J*=7,6 1H),7.92-7.69 (m, 2H), 7.63-7.48 (m, 3H), 7.46 (d, *J* = 456.6 Hz, 1H), 1.19 (d, *J* = 16.6 Hz, 9H).
^{31}P NMR (81 MHz, $CDCl_3$) δ: 52.84
MS (EI): m/z = 232 $[M]^+$
Anal. Calcd. for C14H17OP: C, 72.40; H, 7.38. Found: C, 72.05; H, 7.48.

S2. NMR-Spectra

^{31}P spectrum of *tert*-butyl-(4-methoxyphenyl)phosphinothioic acid (1a)

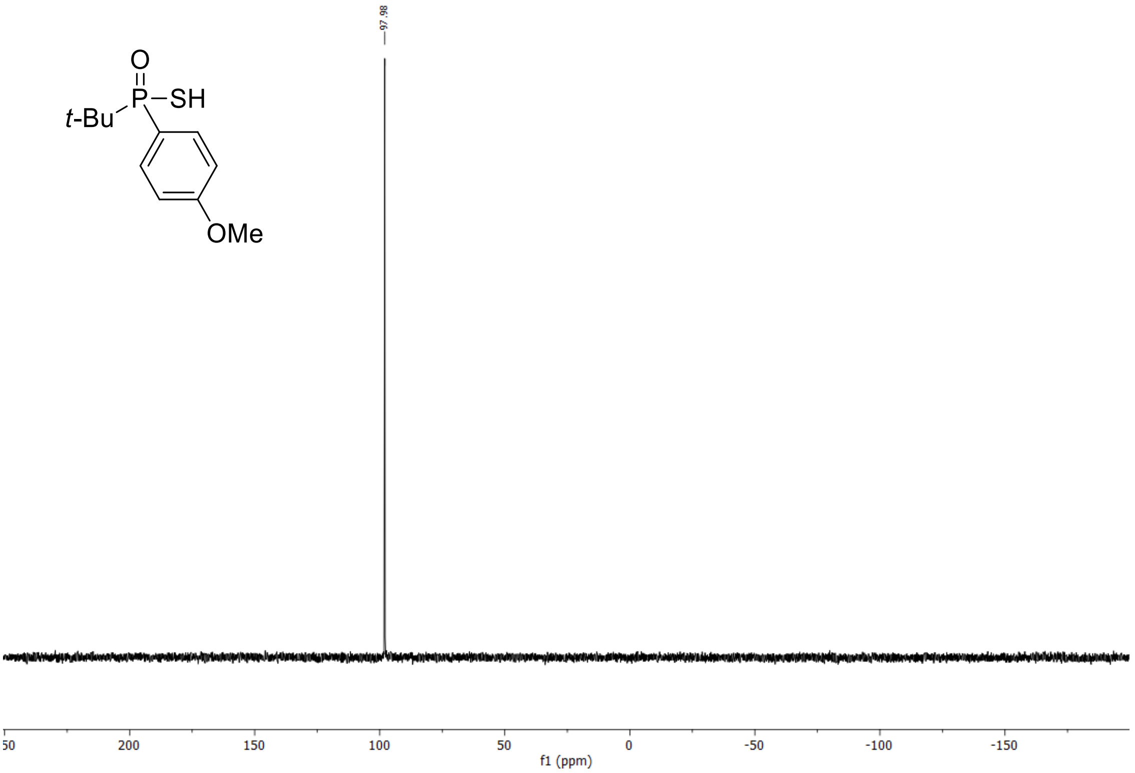

^{1}H spectrum of *tert*-butyl-(4-methoxyphenyl)phosphinothioic acid (1a)

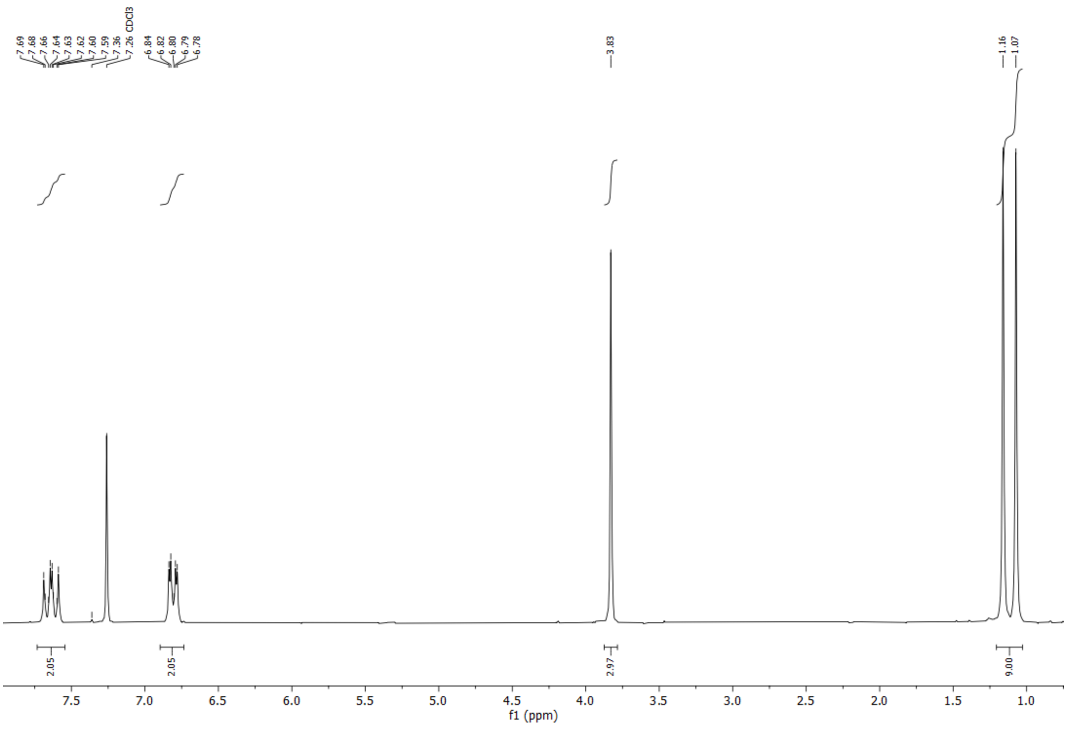

^{13}C spectrum of *tert*-butyl-(4-methoxyphenyl)phosphinothioic acid (1a)

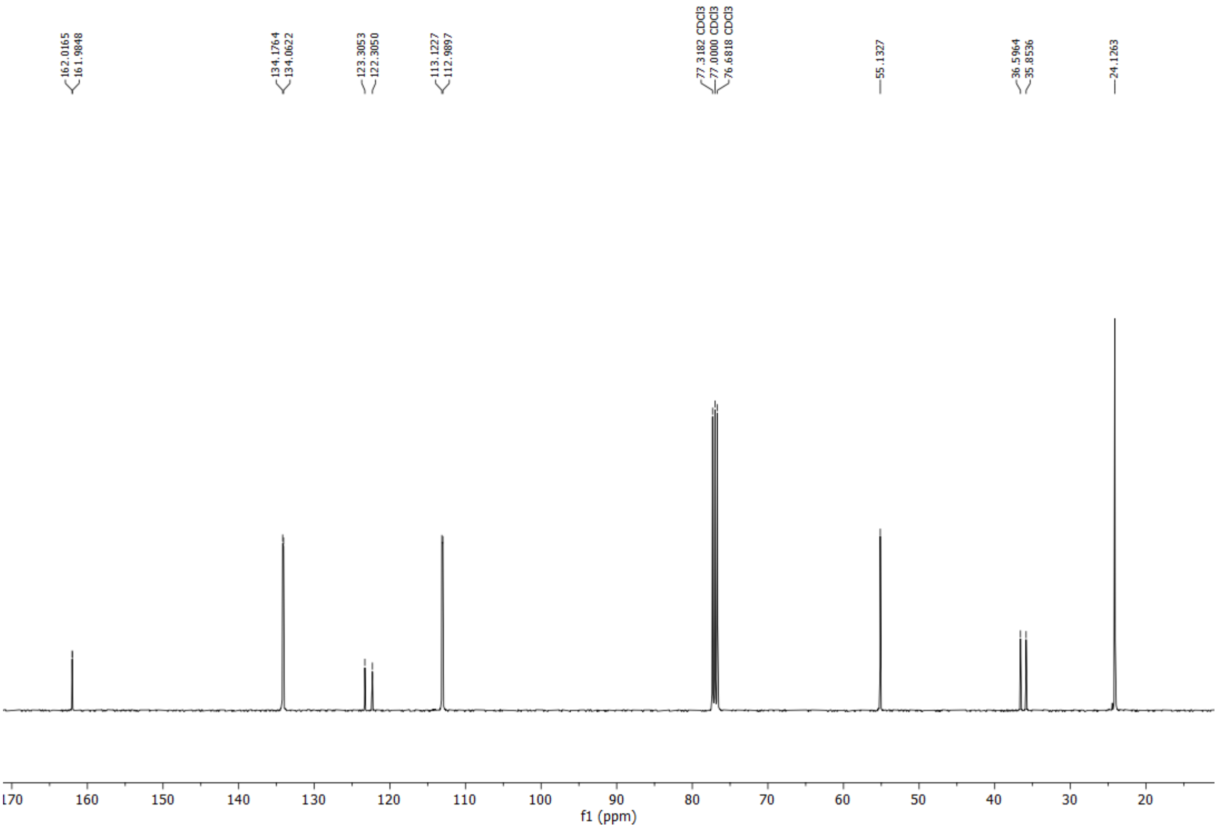

^{31}P spectrum of *tert*-butyl-(4-trifluoromethyl)phosphinothioic acid (1b)

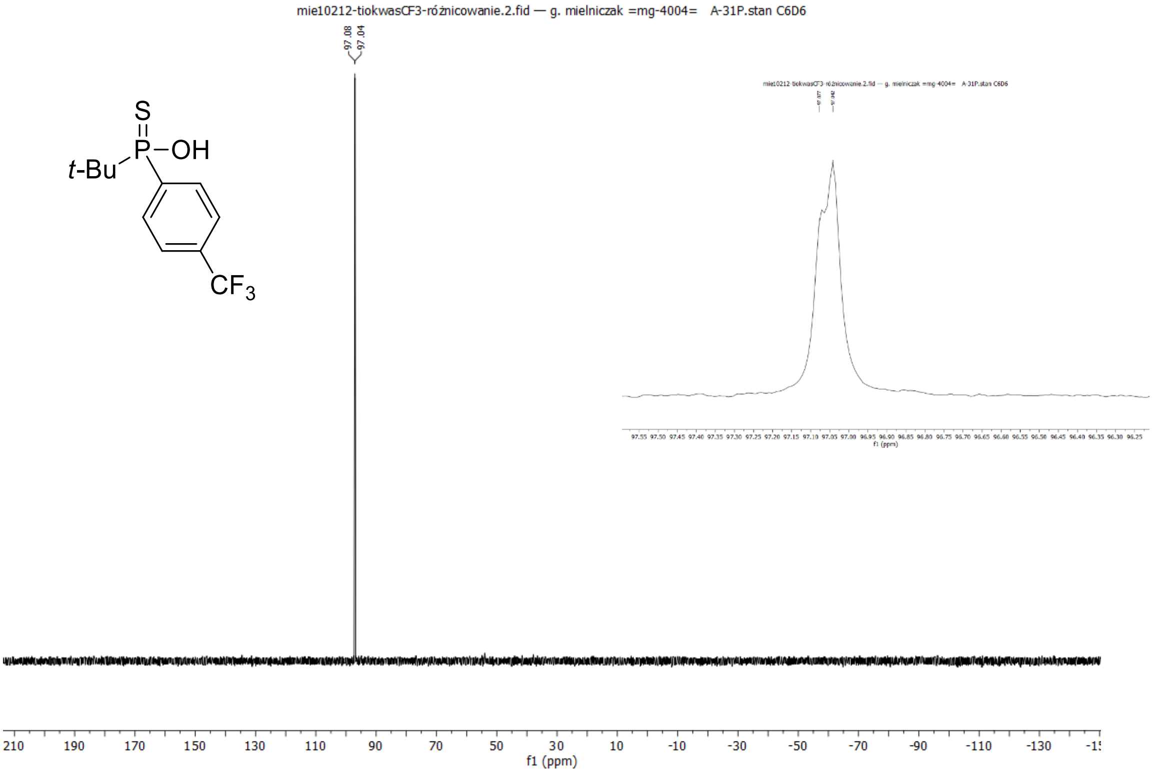

^{19}F spectrum of *tert*-butyl-(4-trifluoromethyl)phosphinothioic acid (1b)

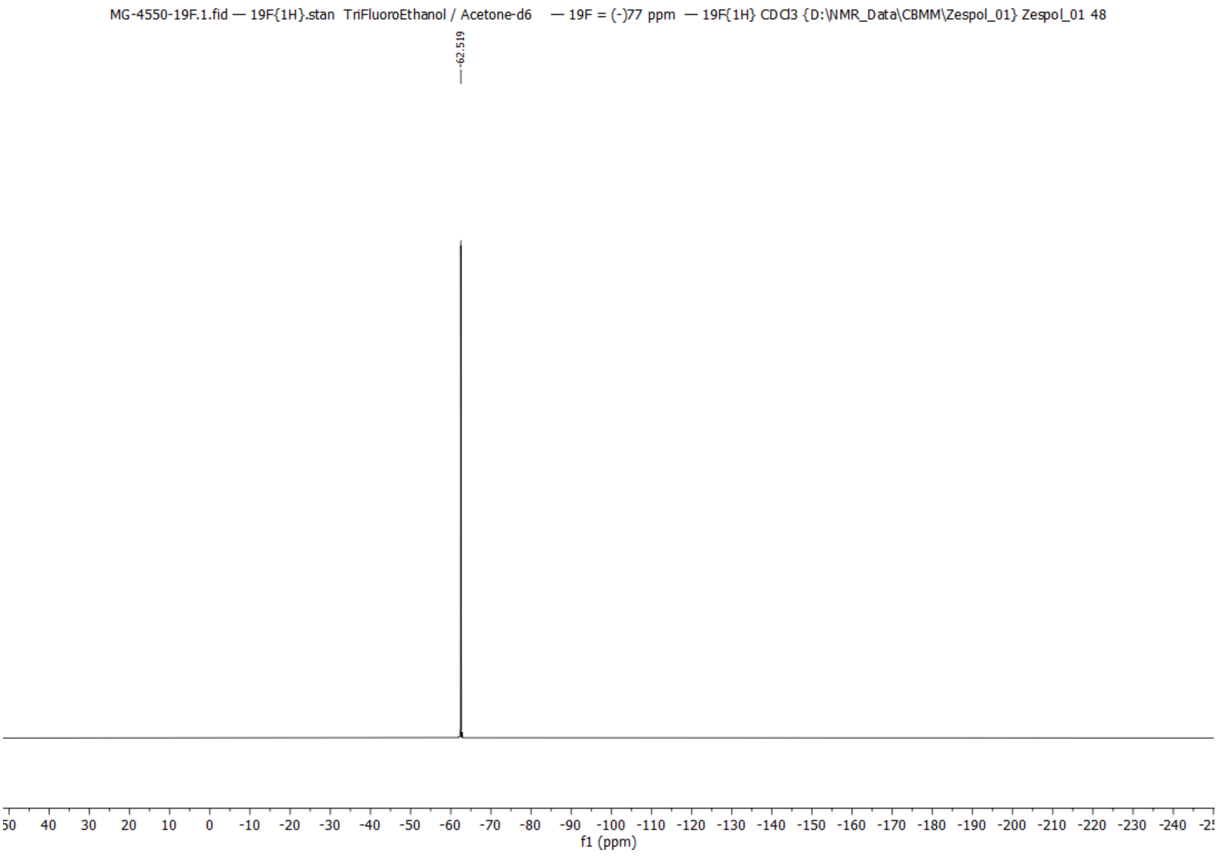

^{1}H spectrum of *tert*-butyl-(4-trifluoromethyl)phosphinothioic acid (1b)

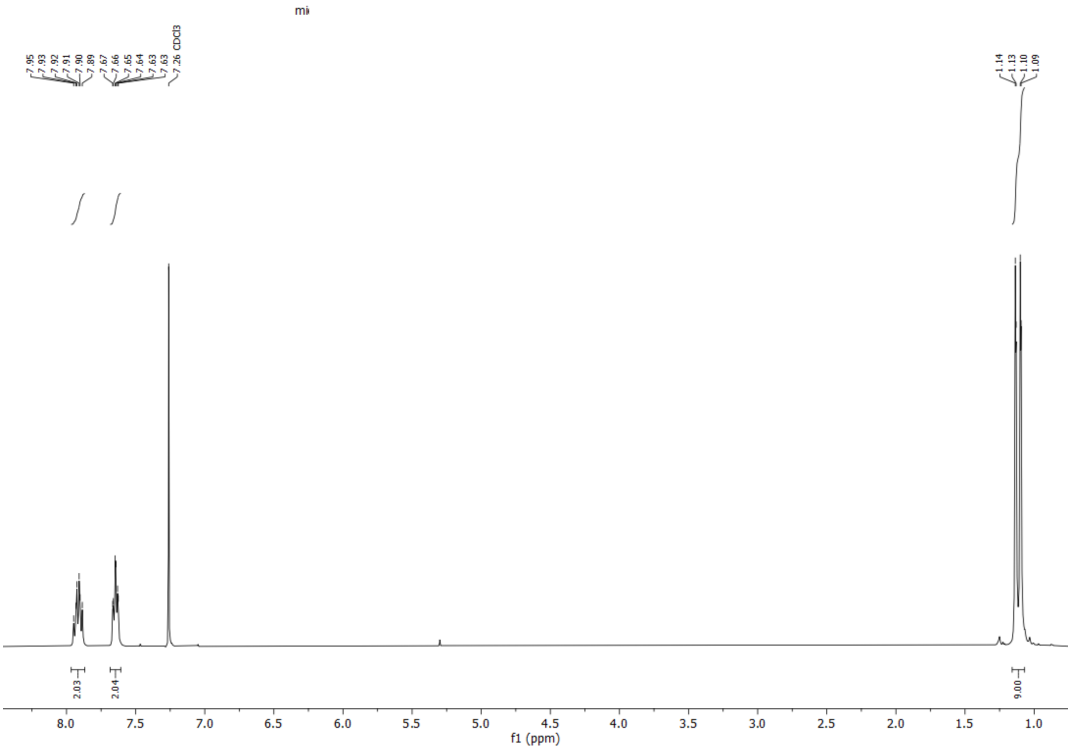

^{13}C spectrum of *tert*-butyl-(4-trifluoromethyl)phosphinothioic acid (1b)

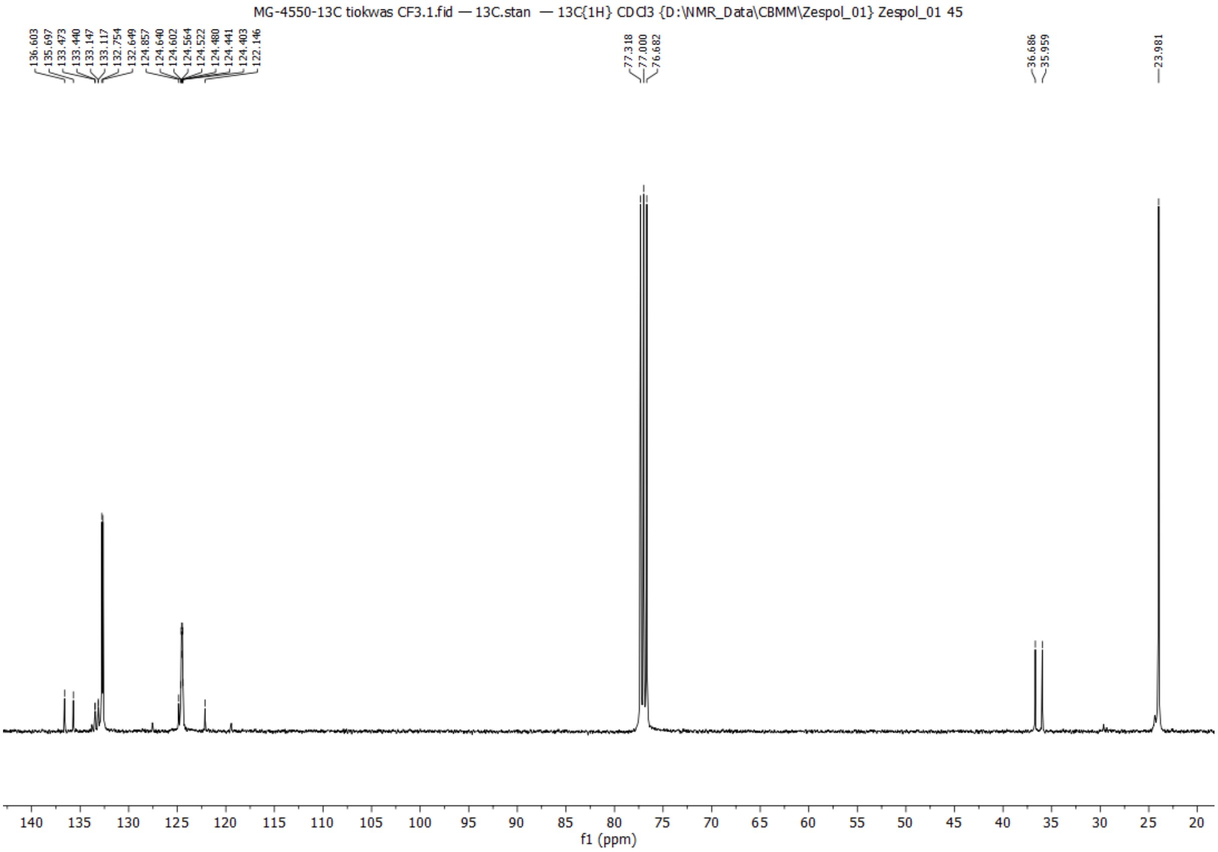

^{31}P spectrum of *tert*-butylphenylphosphinothioic acid (1c)

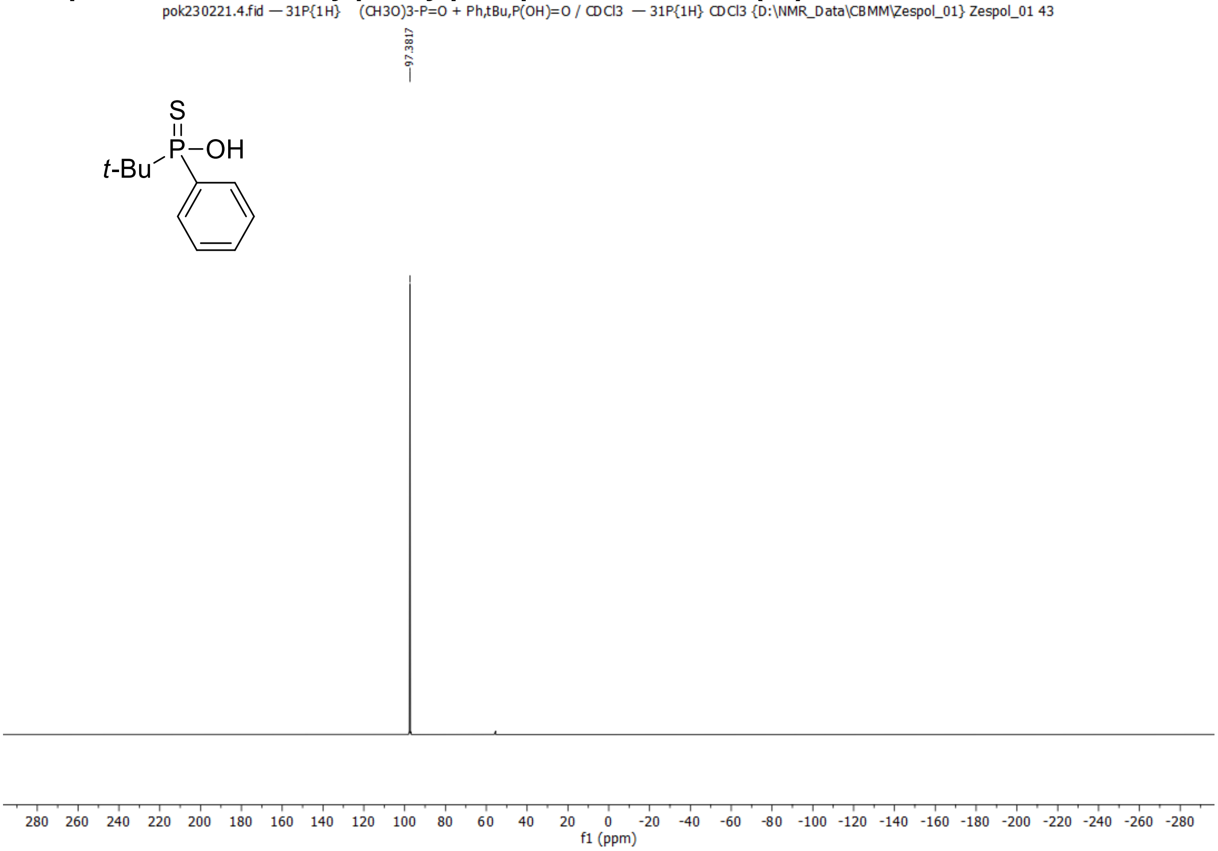

^{1}H spectrum of *tert*-butylphenylphosphinothioic acid (1c)

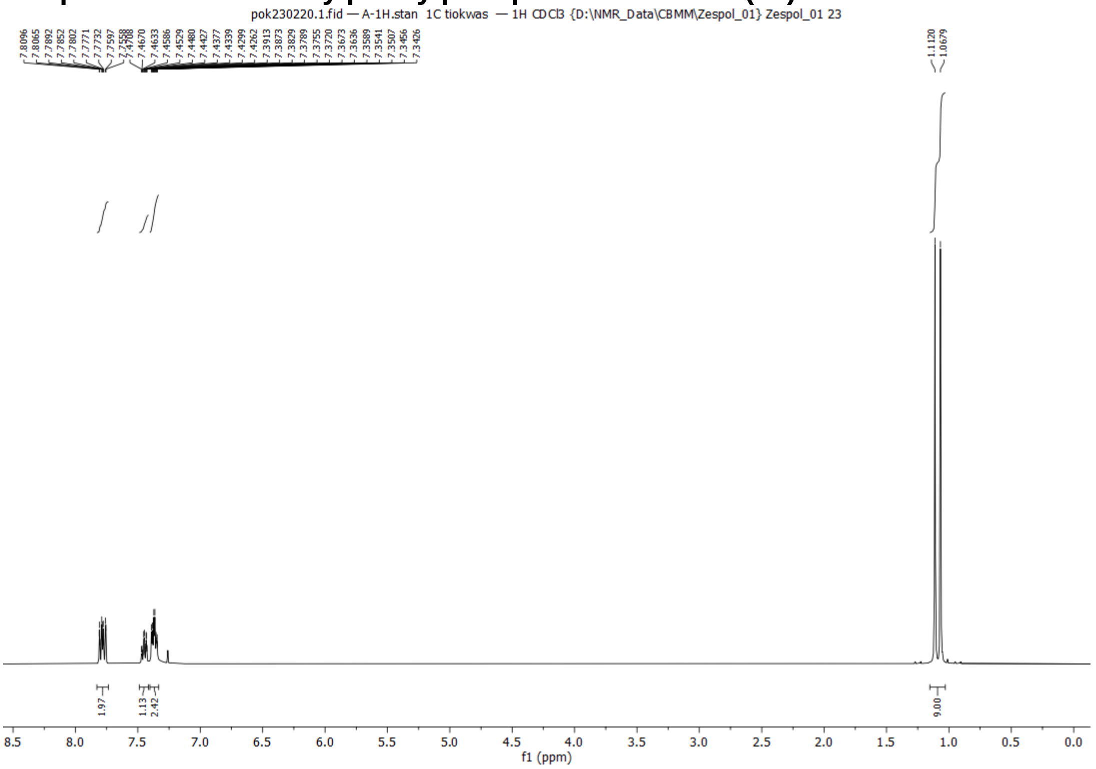

^{13}C spectrum of *tert*-butylphenylphosphinothioic acid (1c)

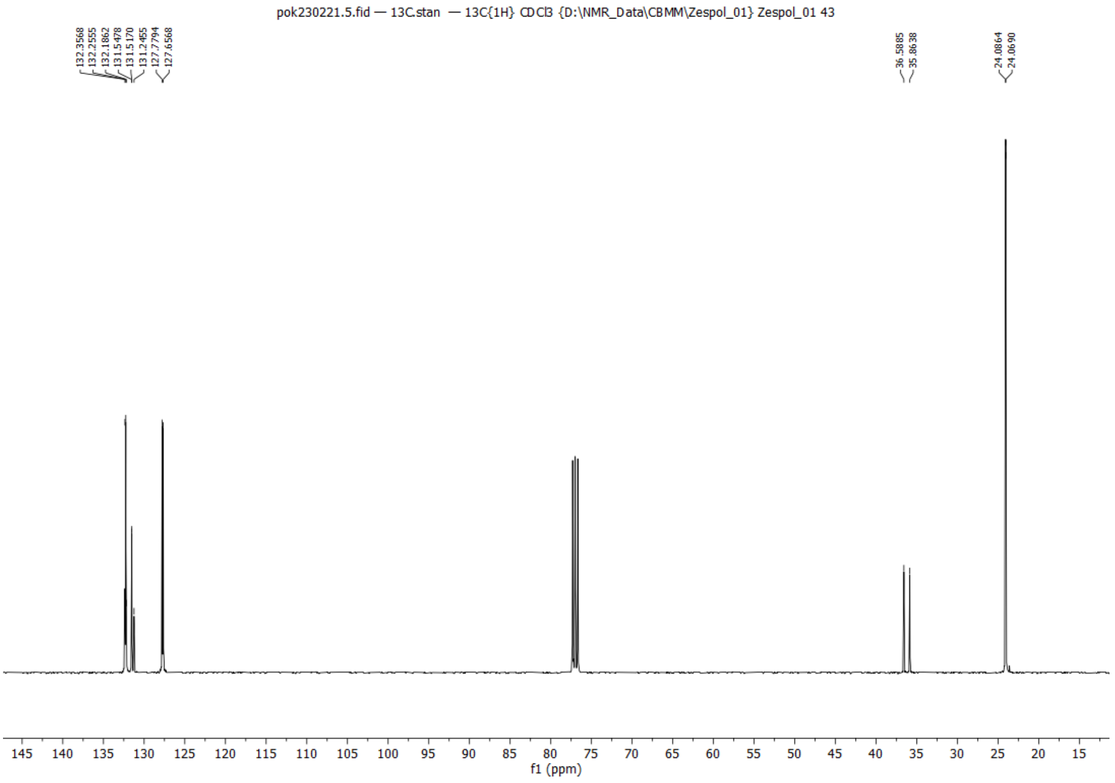

^{31}P spectrum of *tert*-butyl-1-naphtylphosphinothioic acid (1d)

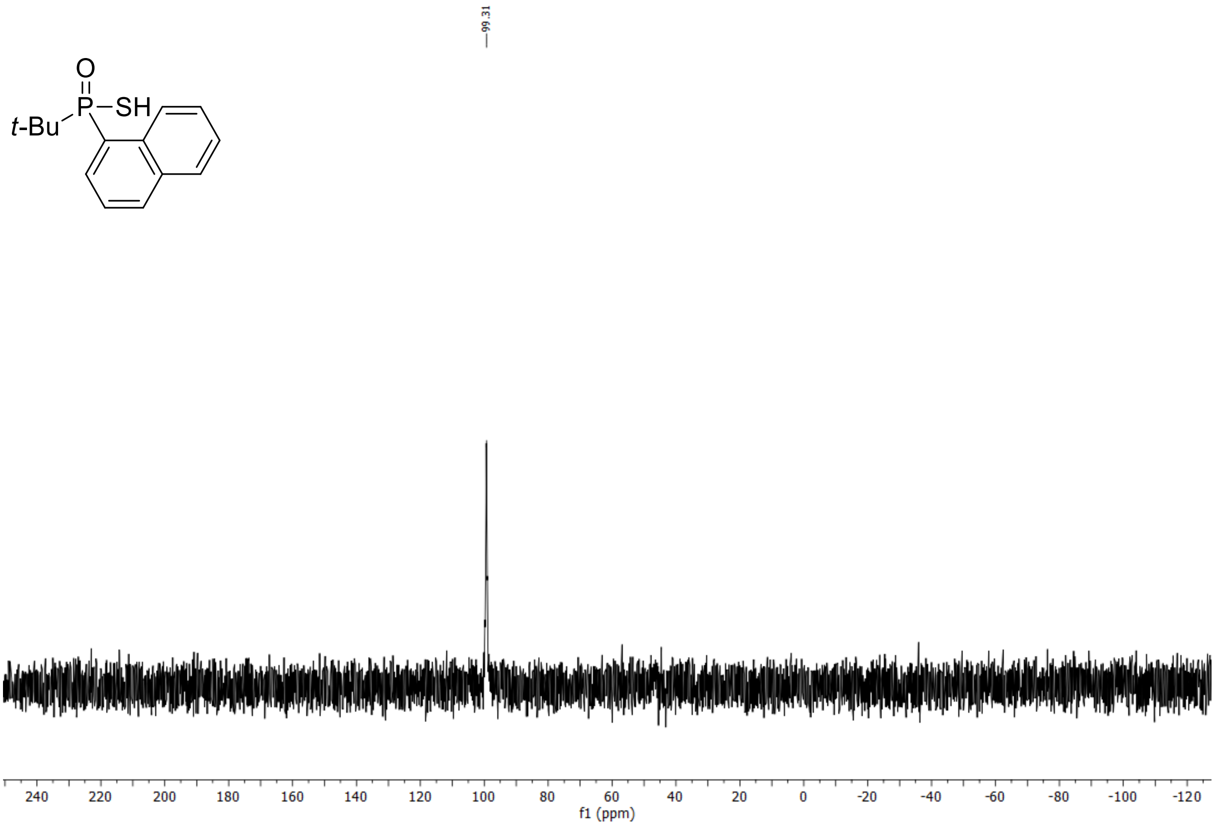

^{1}H spectrum of *tert*-butyl-1-naphtylphosphinothioic acid (1d)

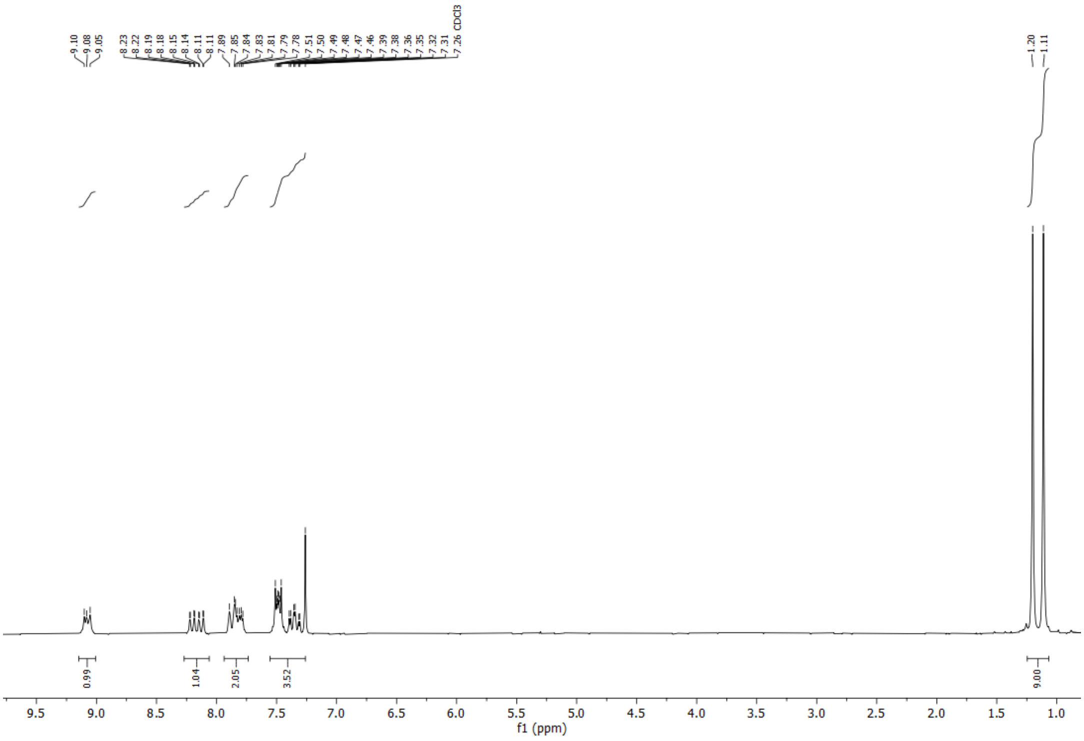

^{13}C spectrum of *tert*-butyl-1-naphtylphosphinothioic acid (1d)

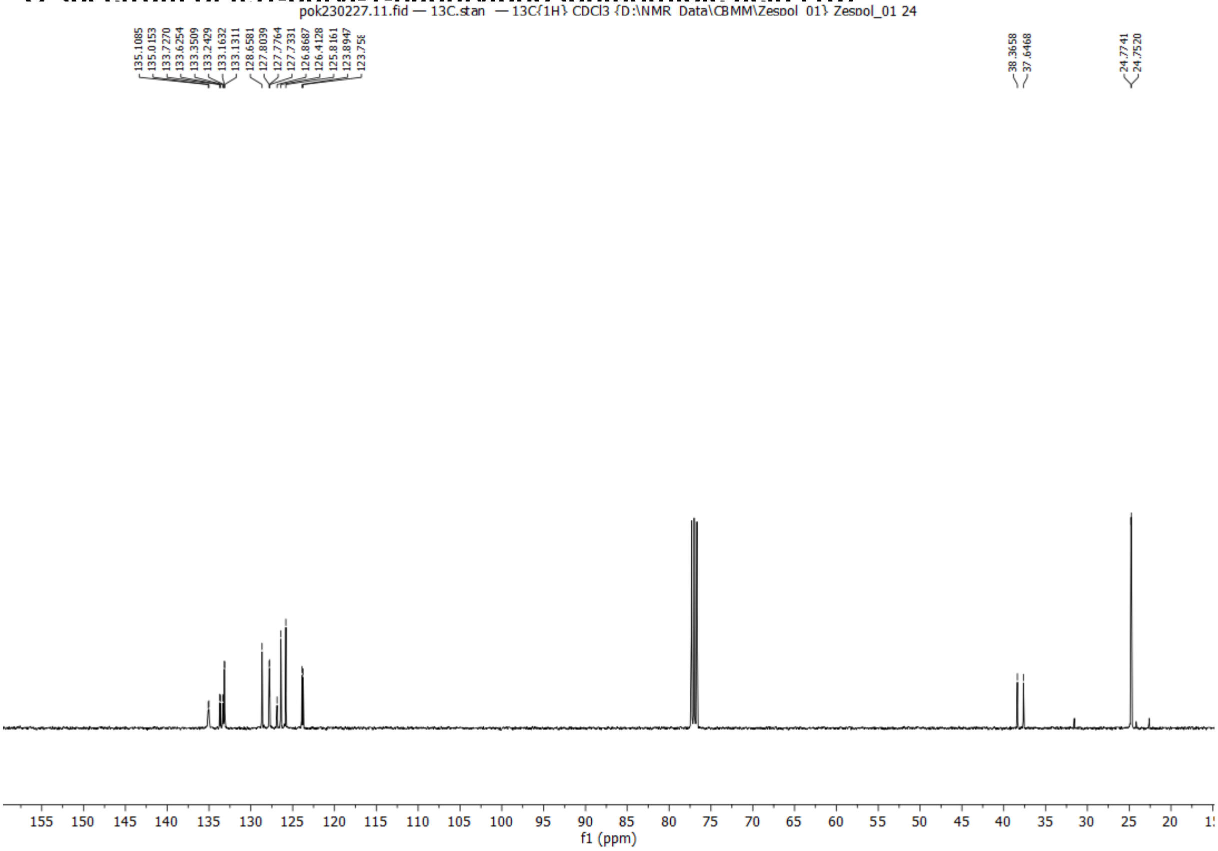

^{31}P spectrum of *tert*-butyl-(4-methoxyphenyl)phosphinoselenoic acid (2a)

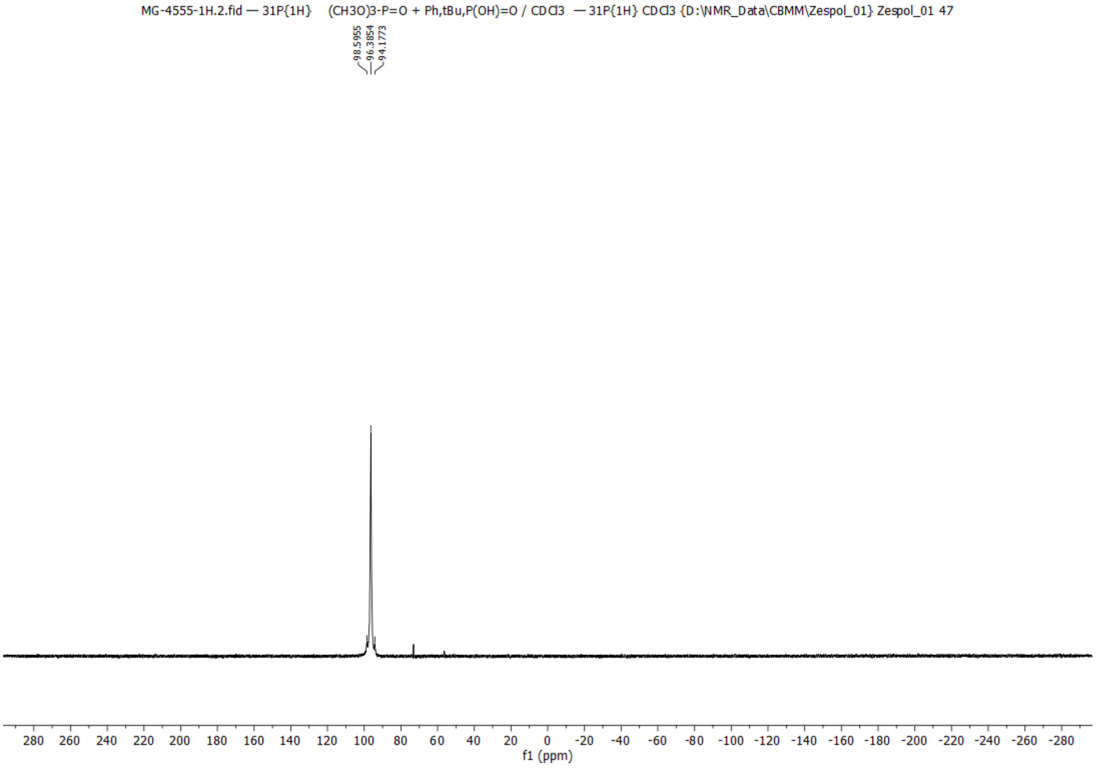

^{1}H spectrum of *tert*-butyl-(4-methoxyphenyl)phosphinoselenoic acid (2a)

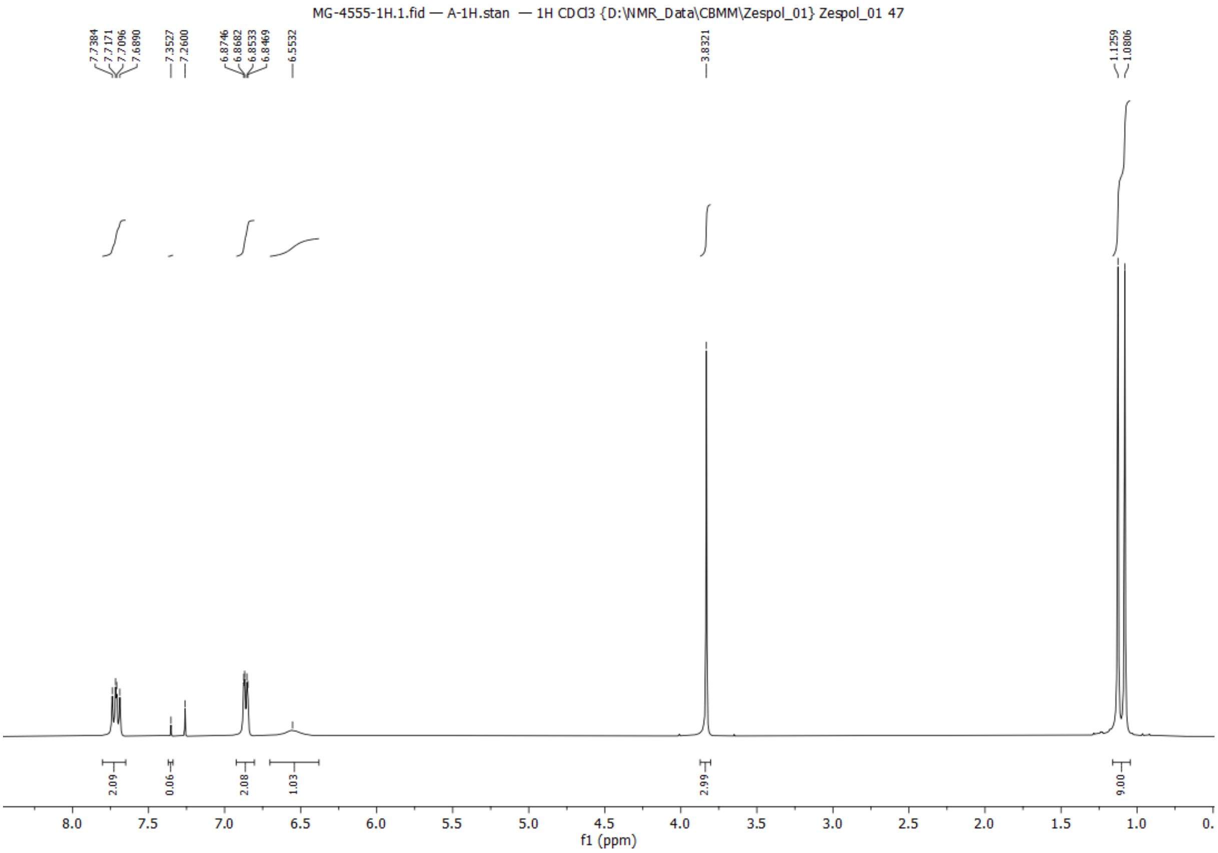

^{13}C spectrum of *tert*-butyl-(4-methoxyphenyl)phosphinoselenoic acid (2a)

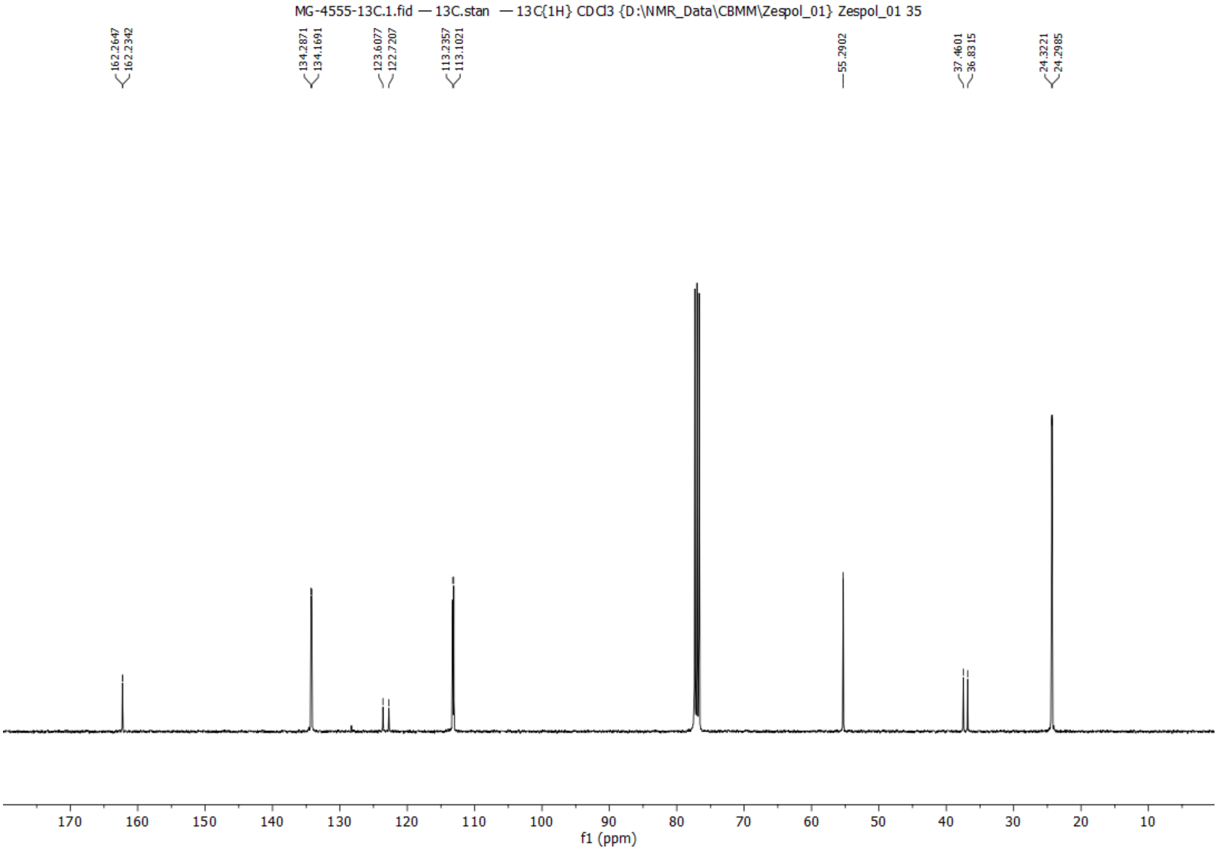

^{31}P spectrum of *tert*-butyl-(4-trifluoromethyl)phosphinoselenoic acid (2b)

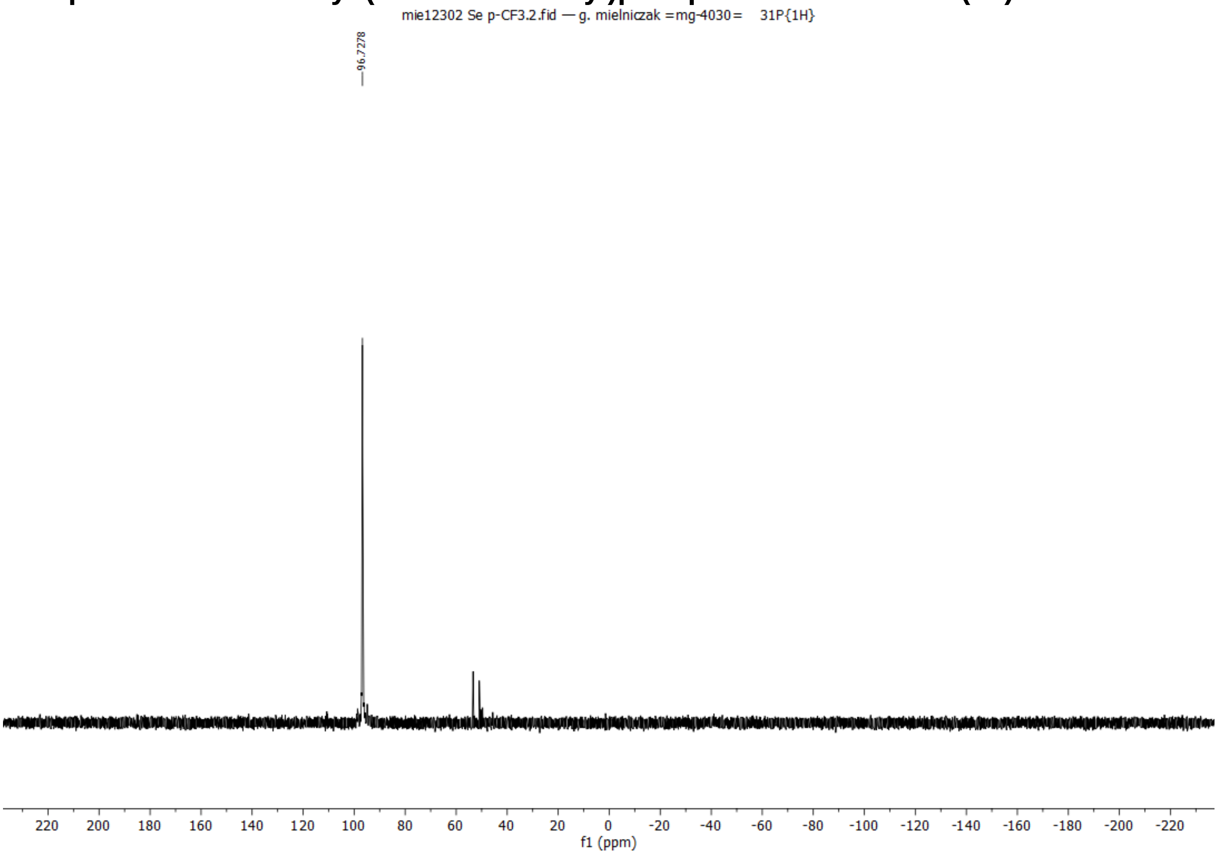

^{1}H spectrum of *tert*-butyl-(4-trifluoromethyl)phosphinoselenoic acid (2b)

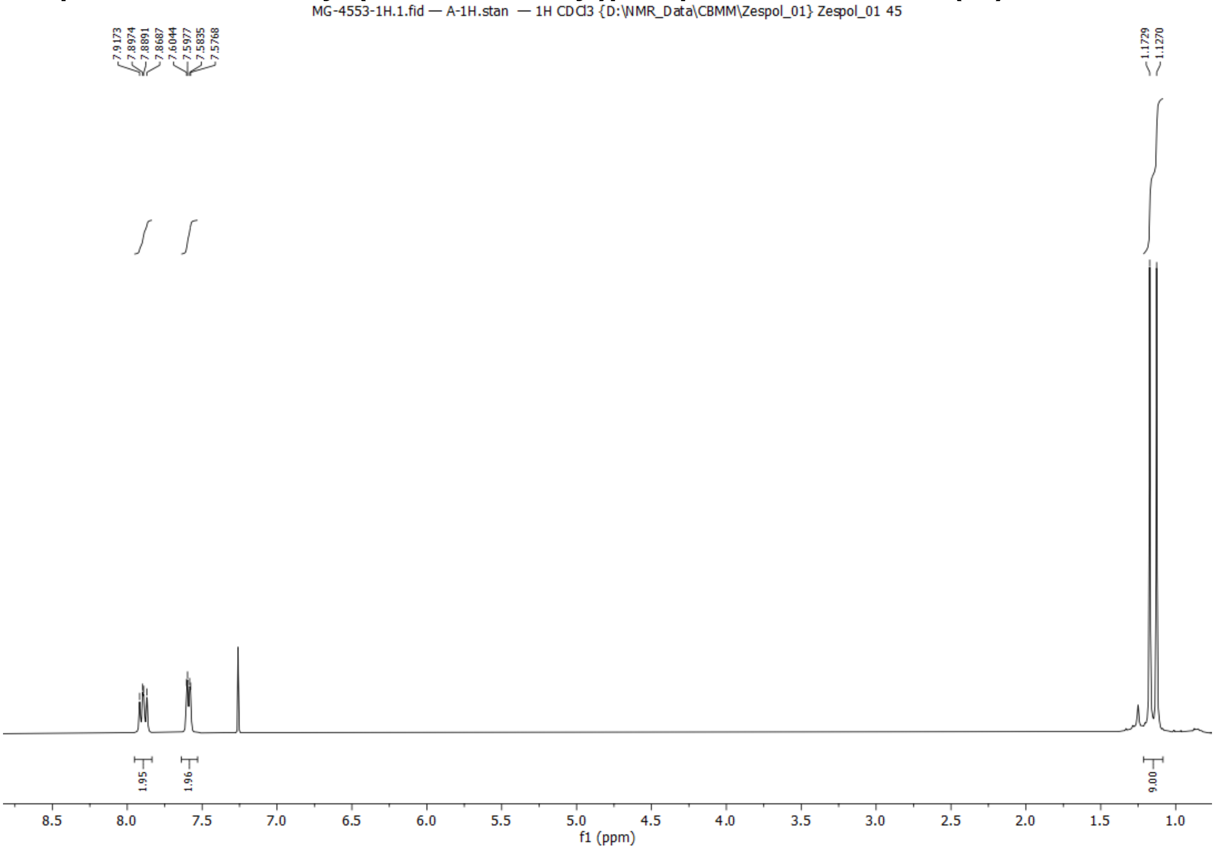

^{13}C spectrum of *tert*-butyl-(4-trifluoromethyl)phosphinoselenoic acid (2b)

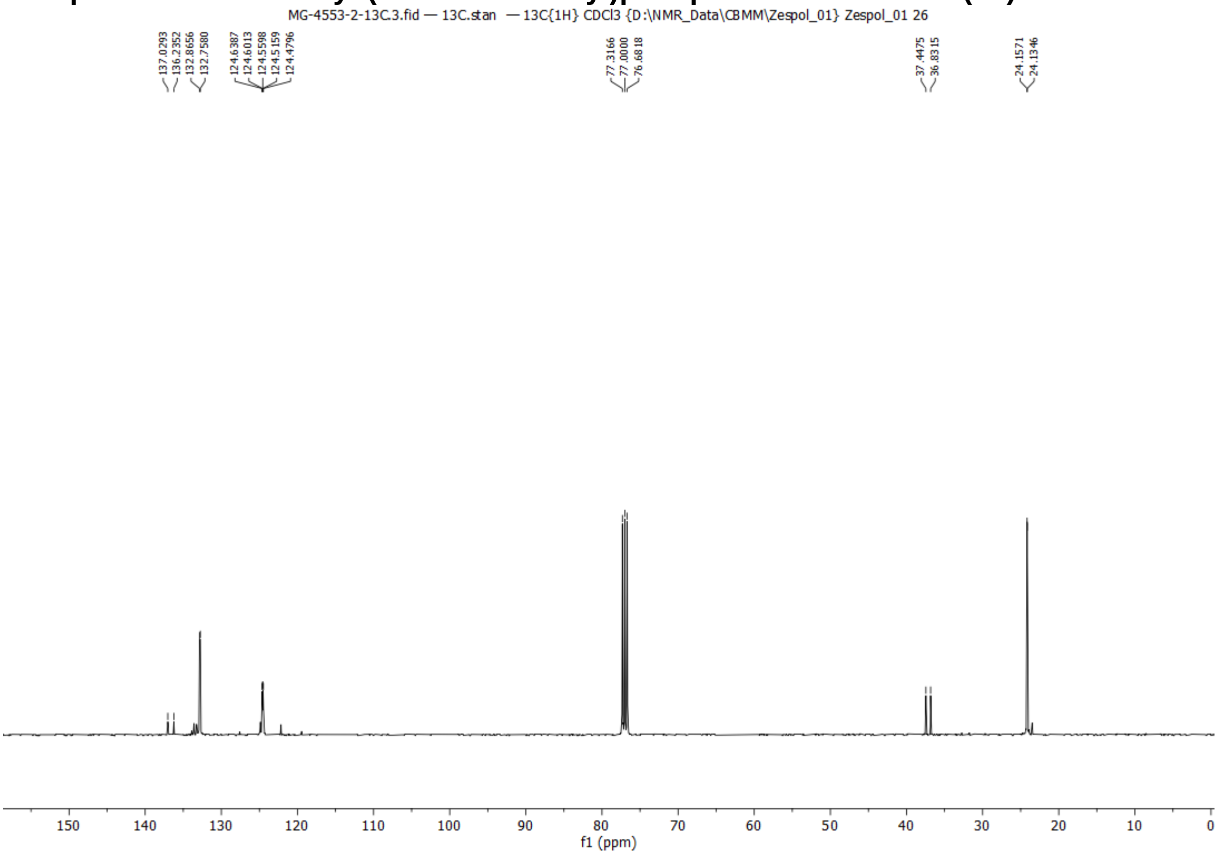

^{13}P spectrum of *tert*-butylphenylphosphinoselenoic acid (2c)

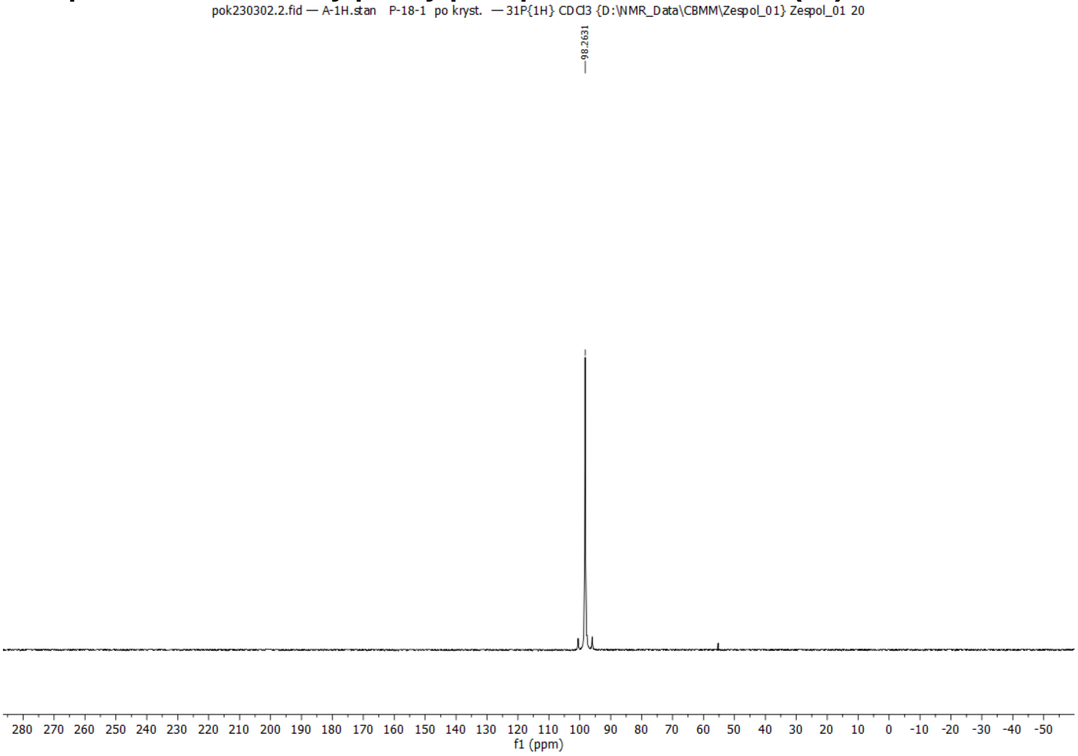

^{1}H spectrum of *tert*-butylphenylphosphinoselenoic acid (2c)

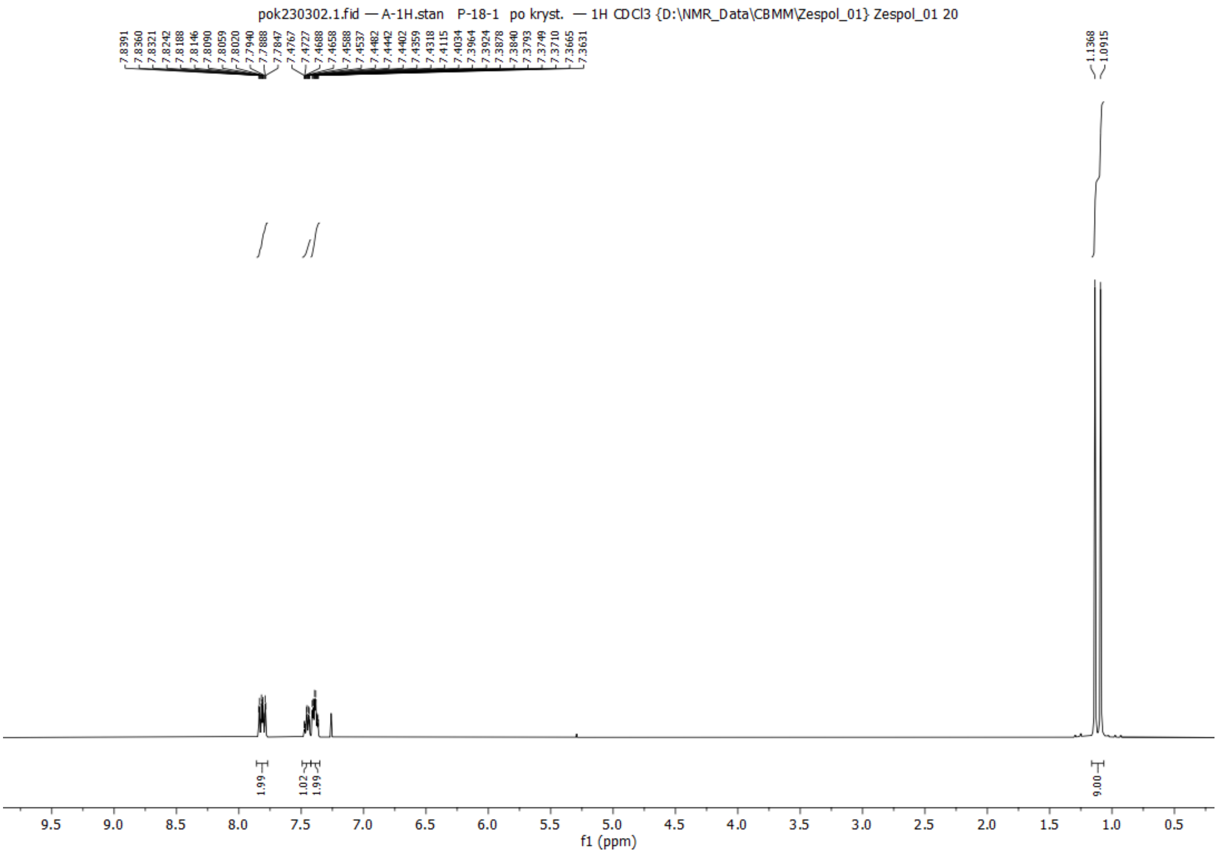

^{13}C spectrum of *tert*-butylphenylphosphinoselenoic acid (2c)

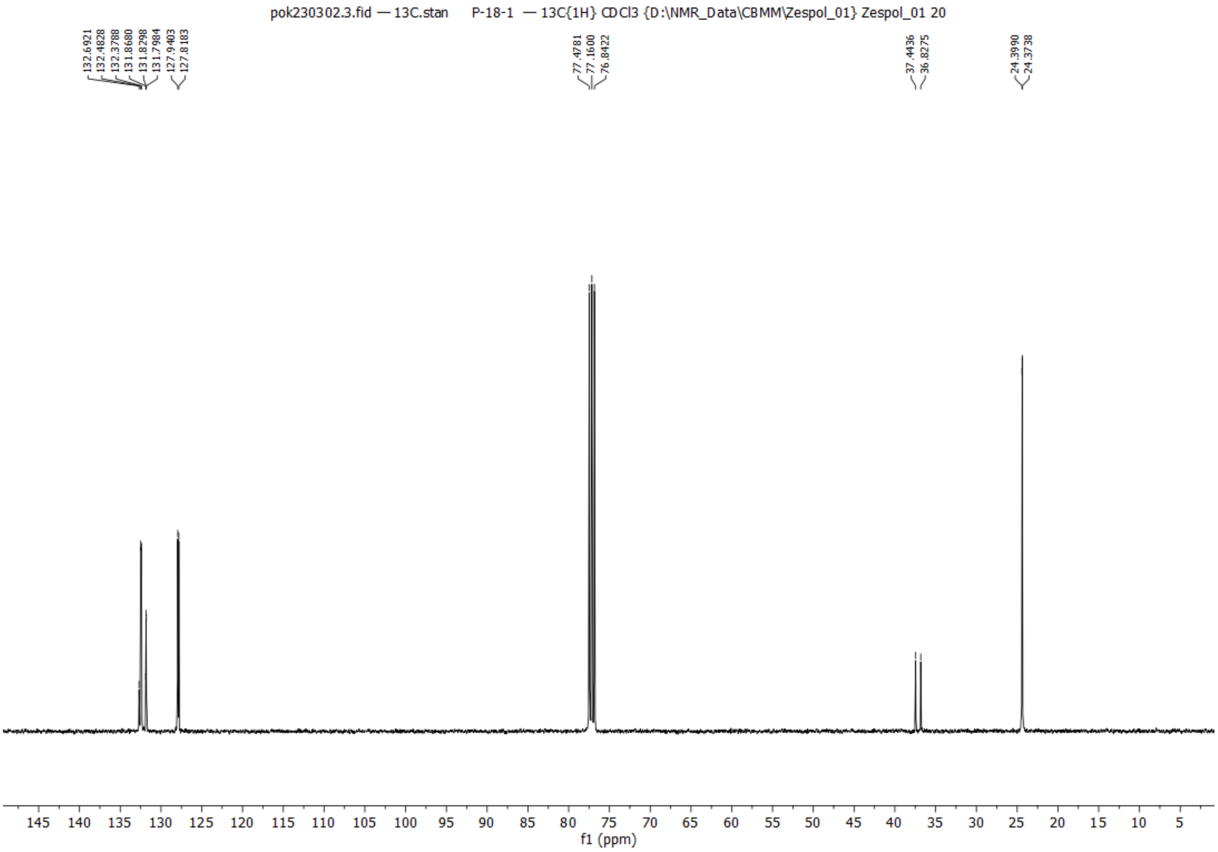

^{31}P spectrum of *tert*-butyl-1-naphtylphosphinoselenoic acid (2d)

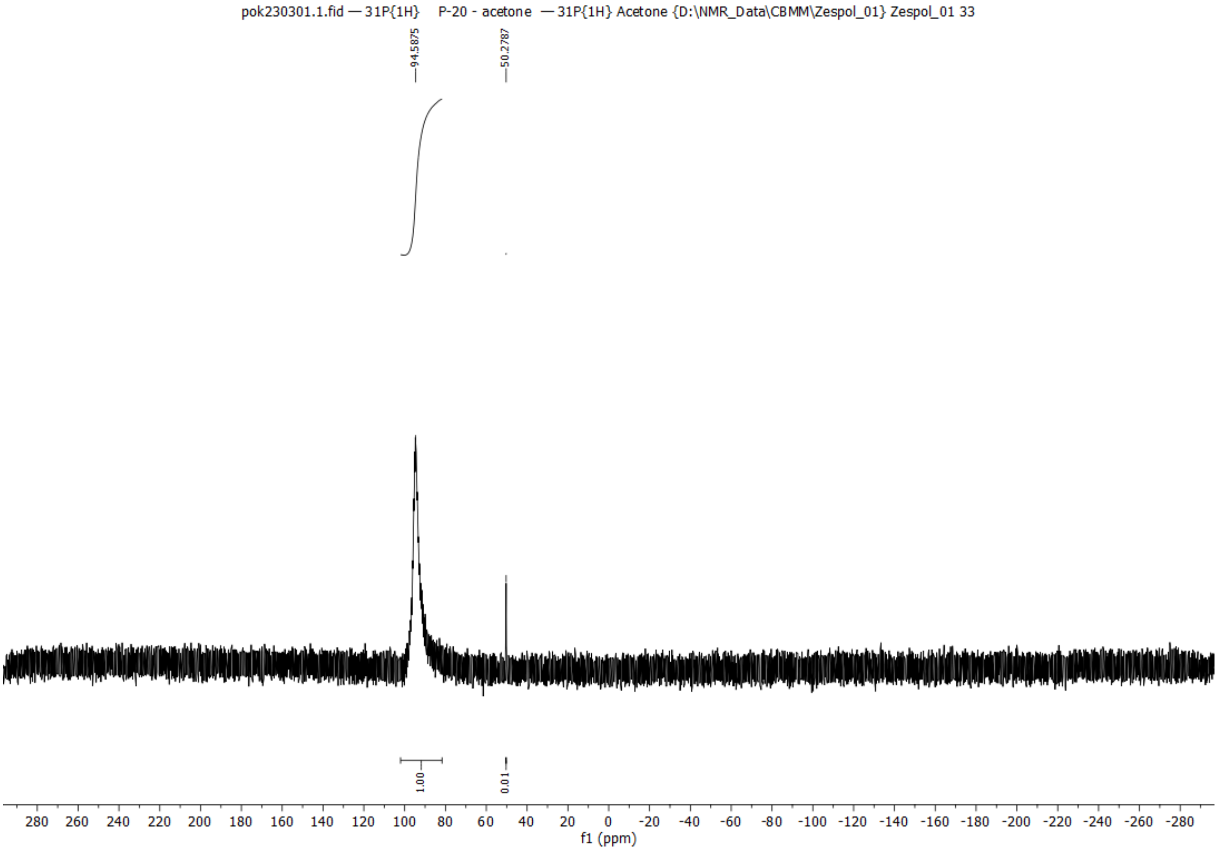

^{1}H spectrum of *tert*-butyl-1-naphtylphosphinoselenoic acid (2d)

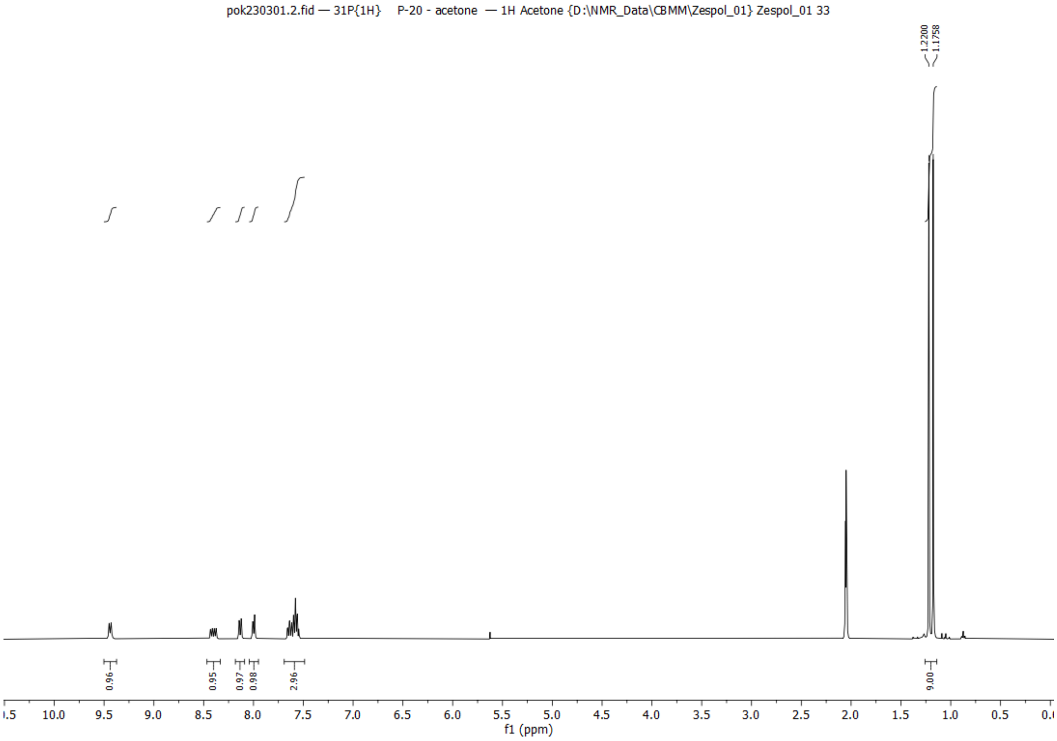

^{13}C spectrum of *tert*-butyl-1-naphtylphosphinoselenoic acid (2d)

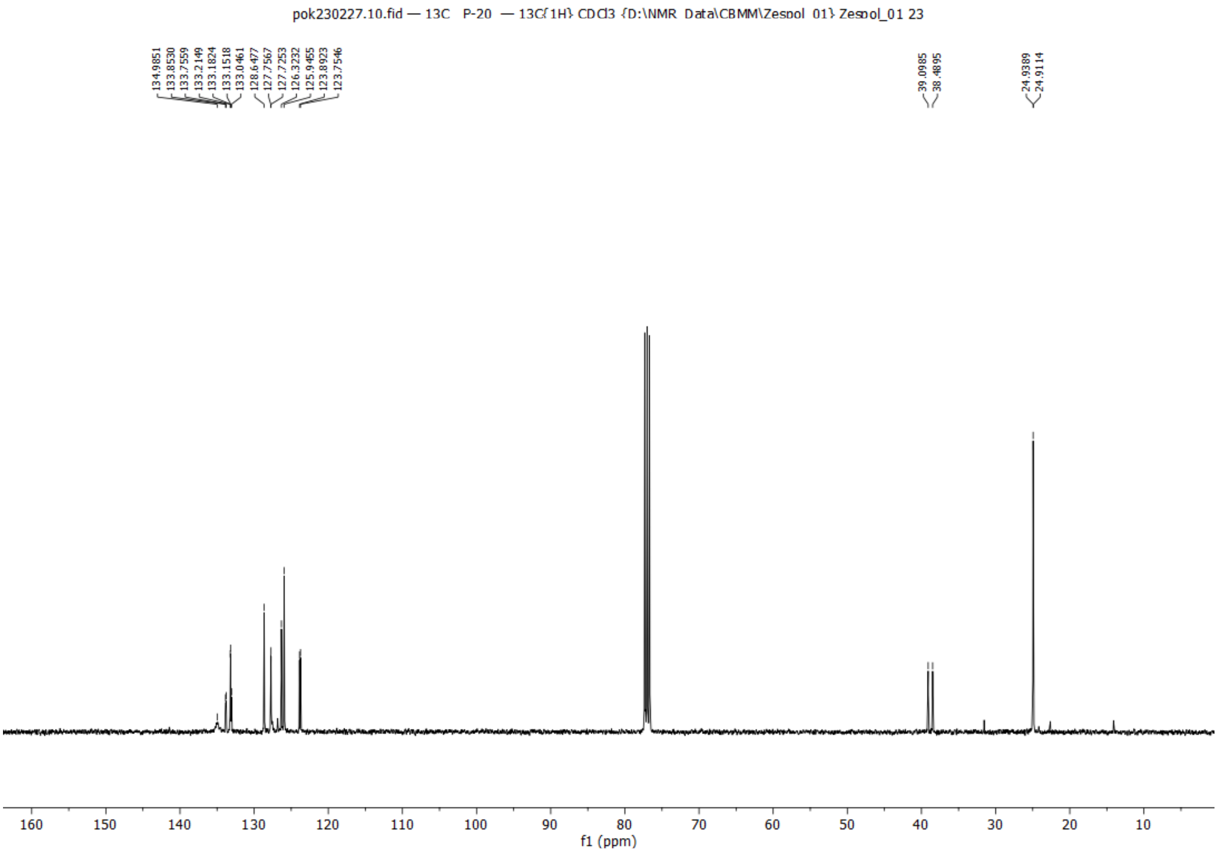

^{31}P spectrum of *tert*-butyl-(4-methoxyphenyl)phosphine oxide (3a)

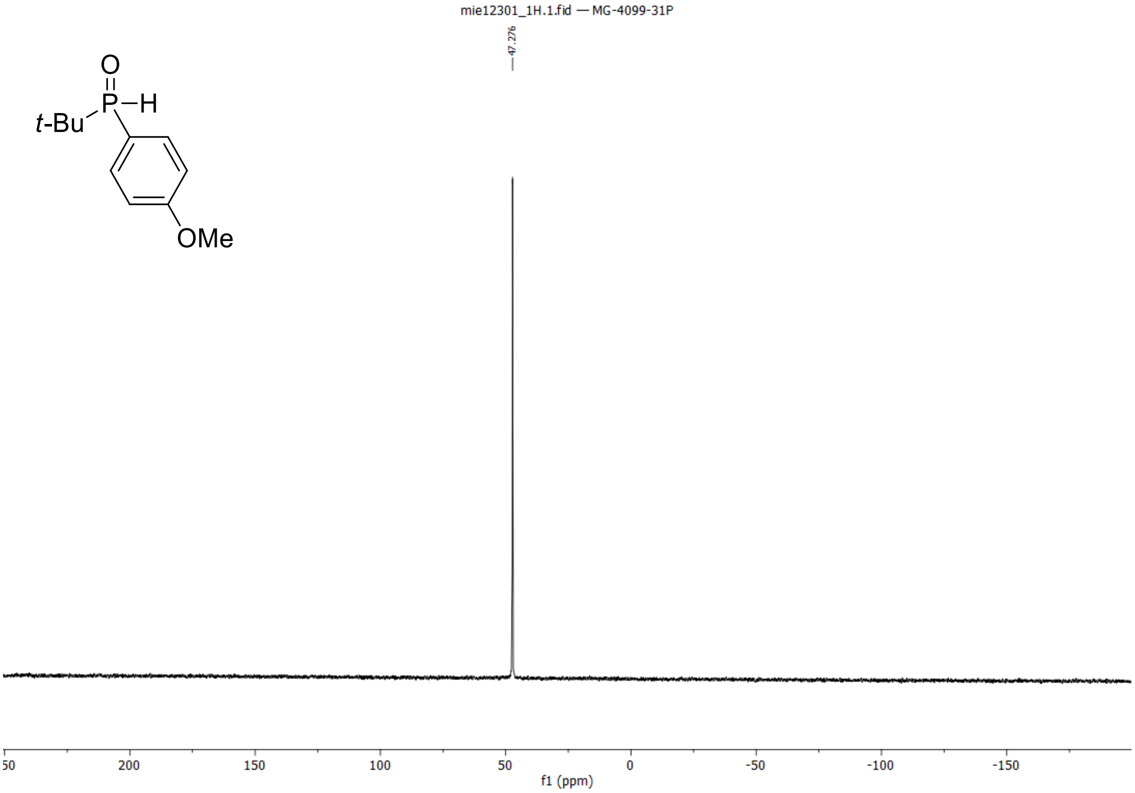

^{1}H spectrum of *tert*-butyl-(4-methoxyphenyl)phosphine oxide (3a)

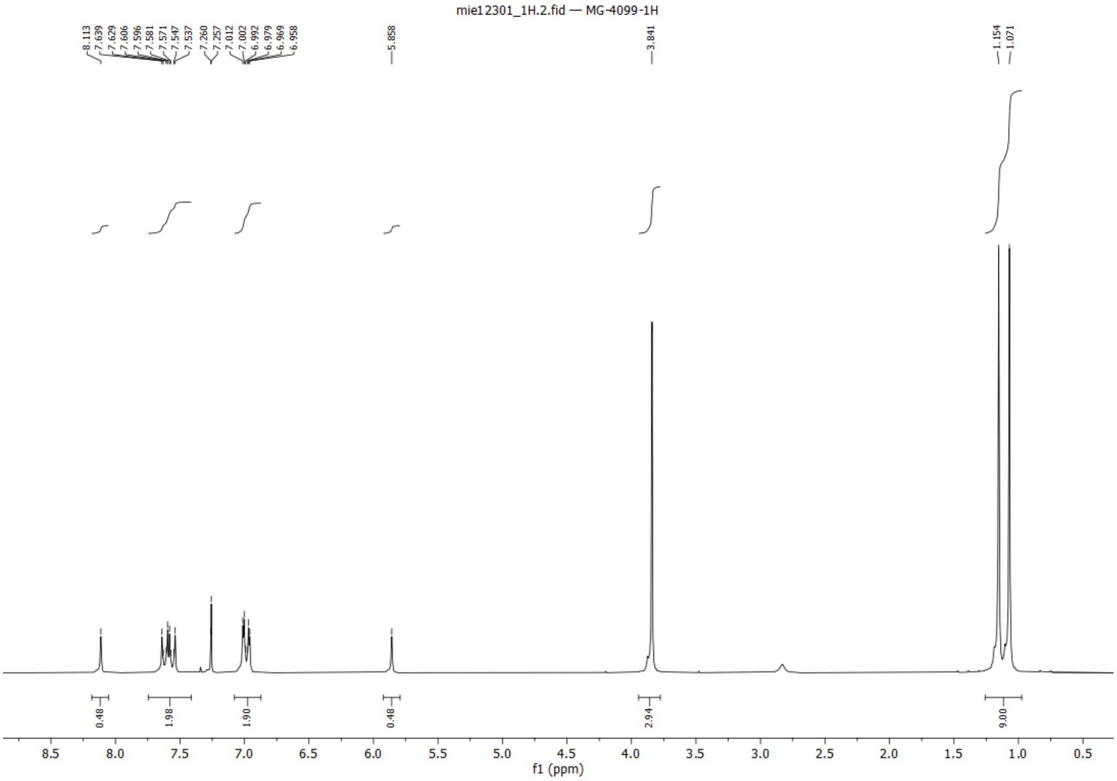

^{31}P spectrum of *tert*-butyl-(4-trifluoromethyl)phenyl phosphine oxide (3b)

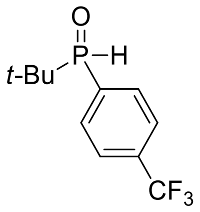

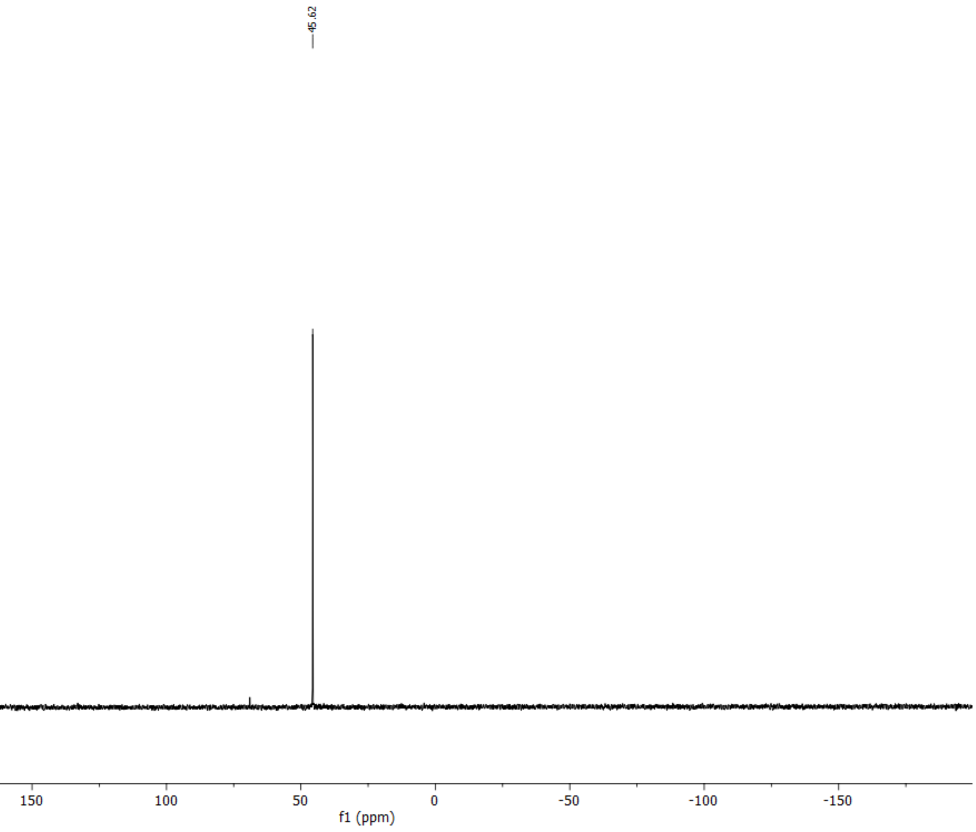

^{1}H spectrum of *tert*-butyl-(4-trifluoromethyl)phenyl phosphine oxide (3b)

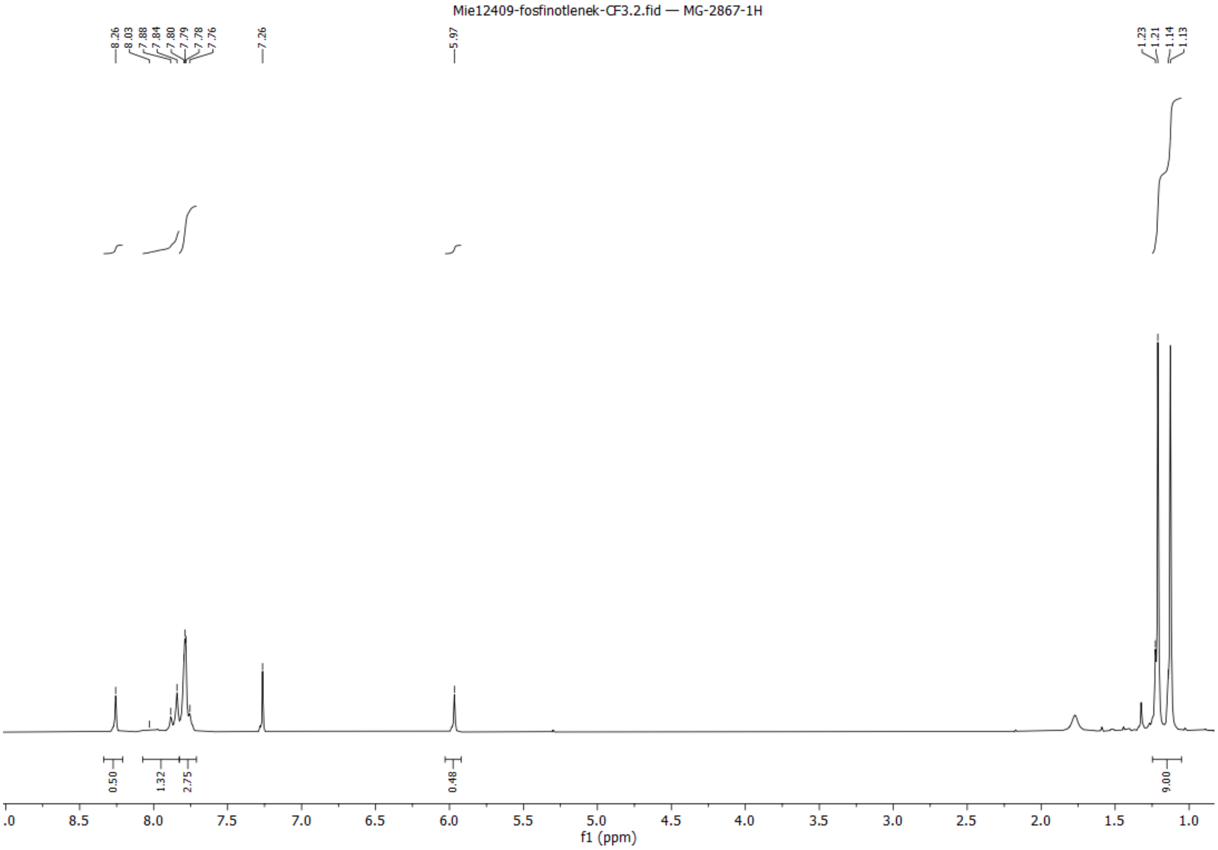

^{31}P spectrum of *tert*-butylphenylphosphine oxide (3c)

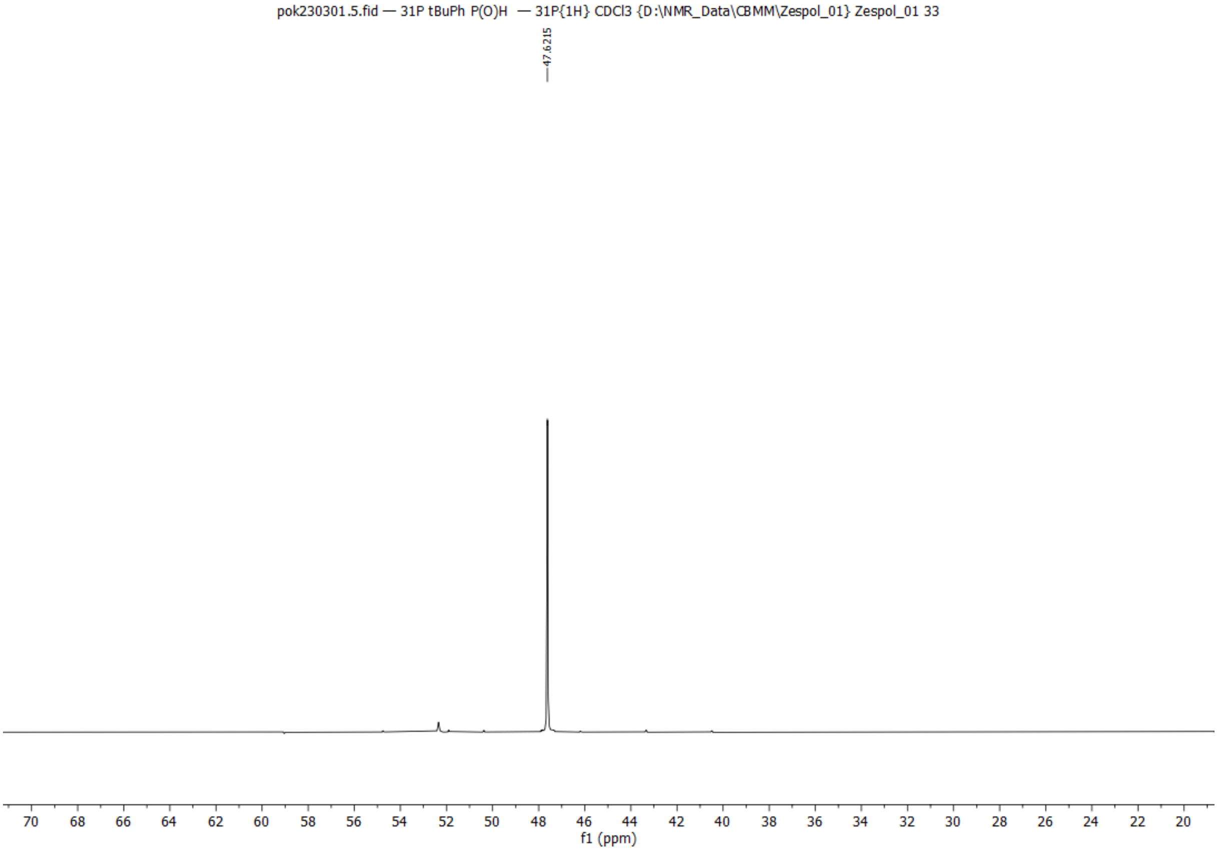

^{1}H spectrum of *tert*-butylphenylphosphine oxide (3c)

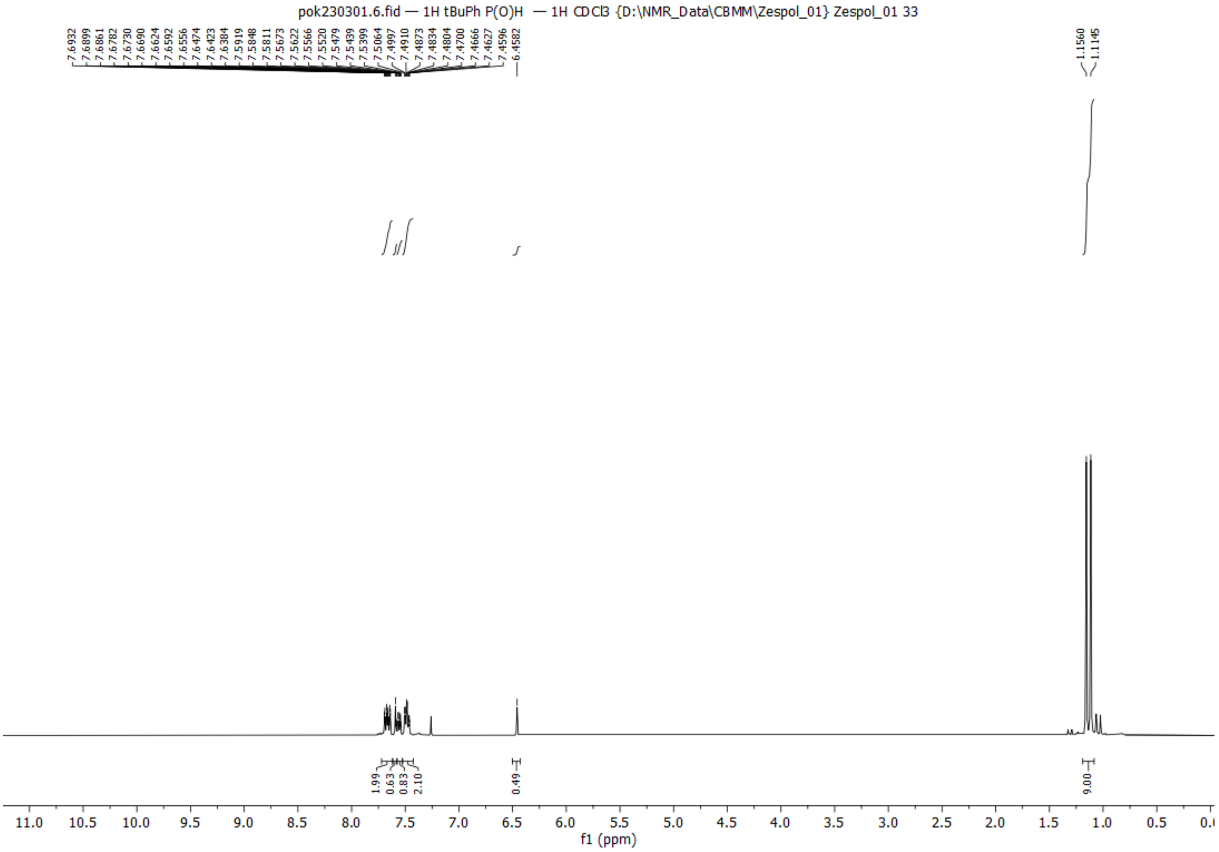

^{31}P spectrum of *tert*-butyl-1-naphthylphosphine oxide (3d)

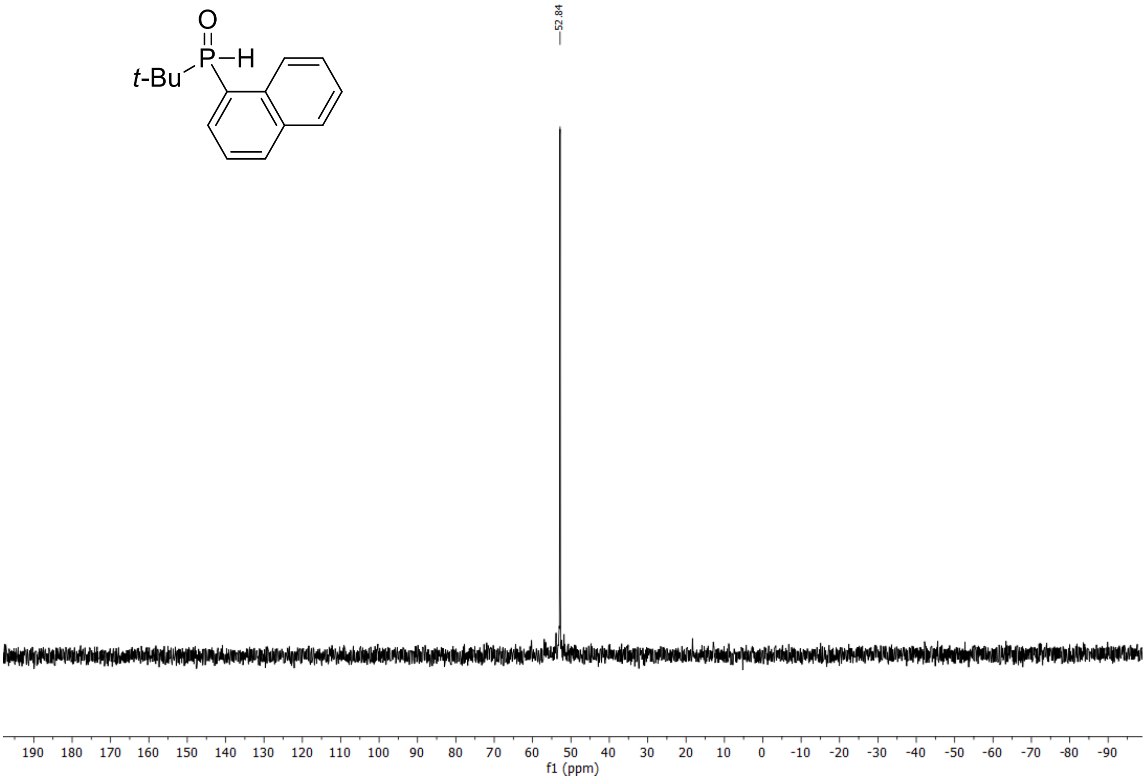

^{1}H spectrum of *tert*-butyl-1-naphthylphosphine oxide (3d)

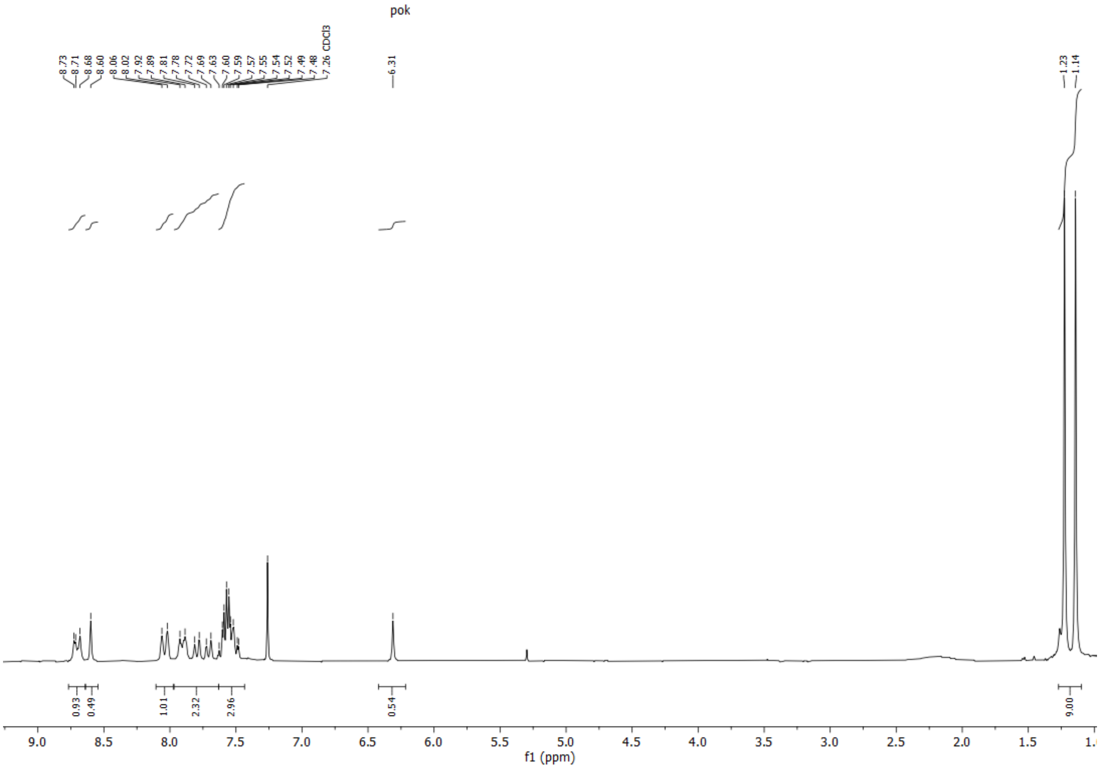

S3. UV-VIS Spectra

UV-VIS spectrum of *tert*-butyl-(4-methoxyphenyl)phosphinothioic acid (1a)

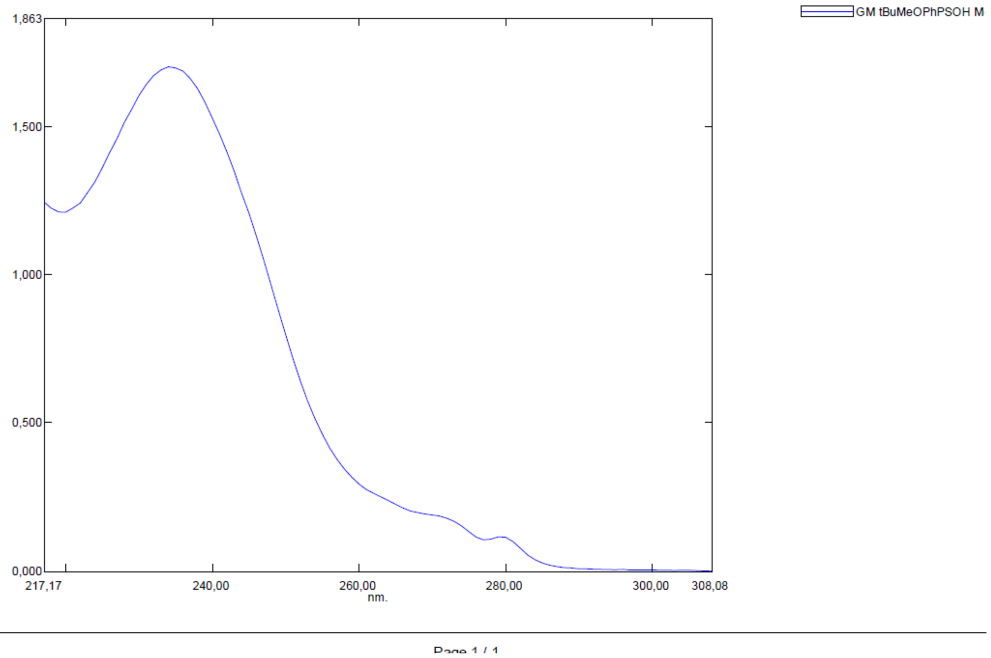

UV-VIS spectrum of *tert*-butyl-(4-trifluoromethyl)phosphinothioic acid (1b)

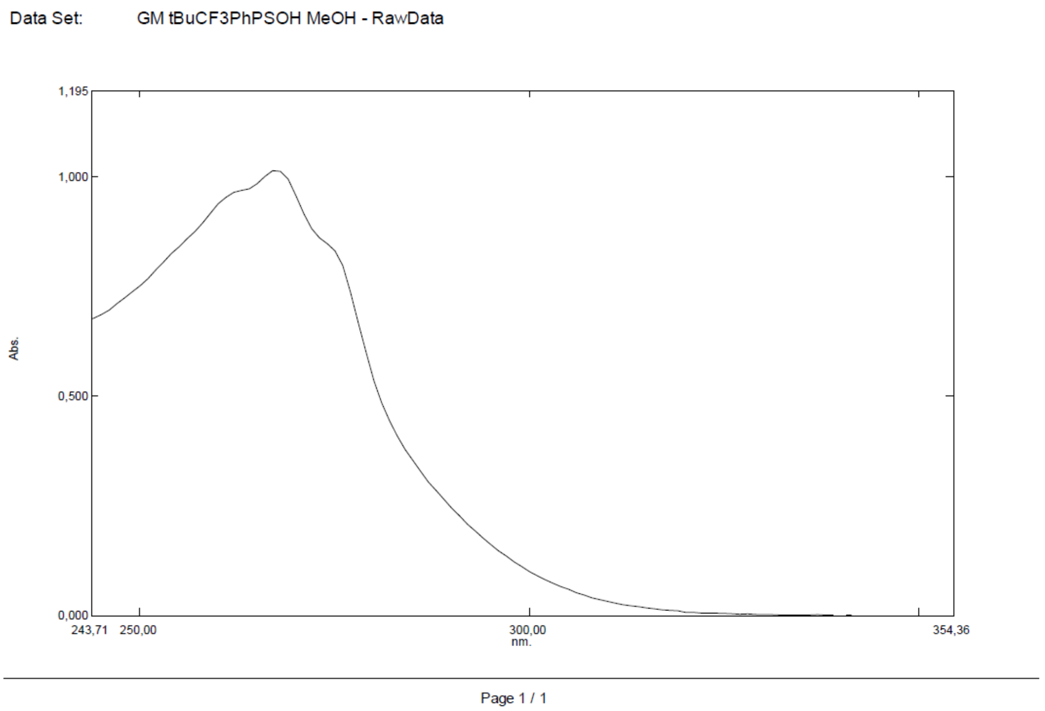

UV-VIS spectrum of *tert*-butyl-1-naphtylphosphinothioic acid (1d)

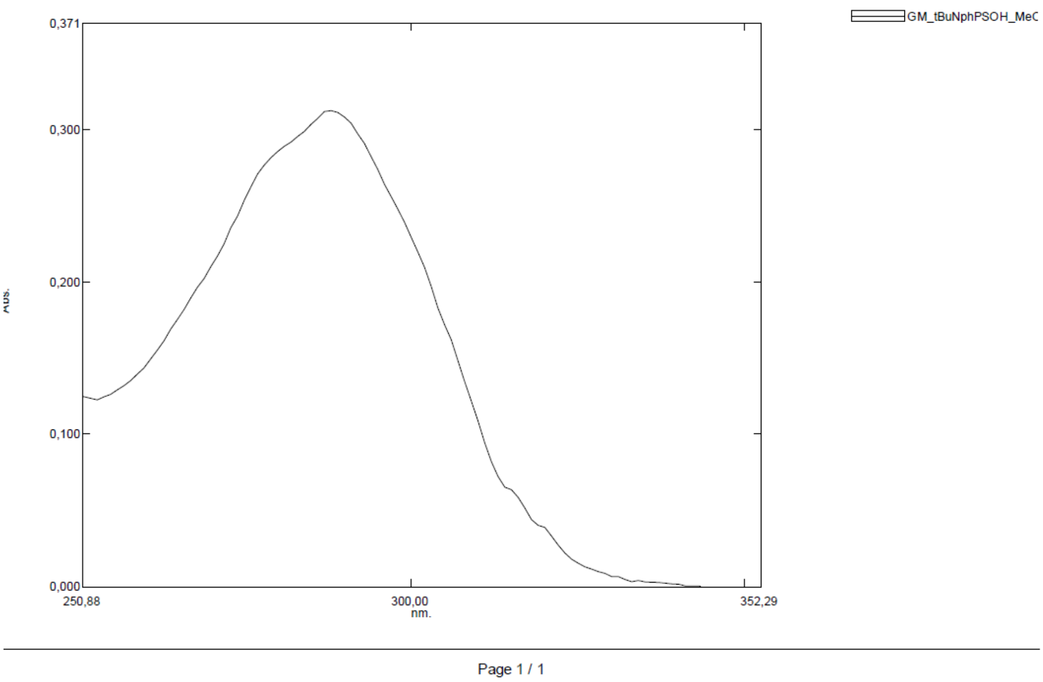

UV-VIS spectrum of *tert*-butyl-(4-methoxyphenyl)phosphinoselenoic acid (2a)

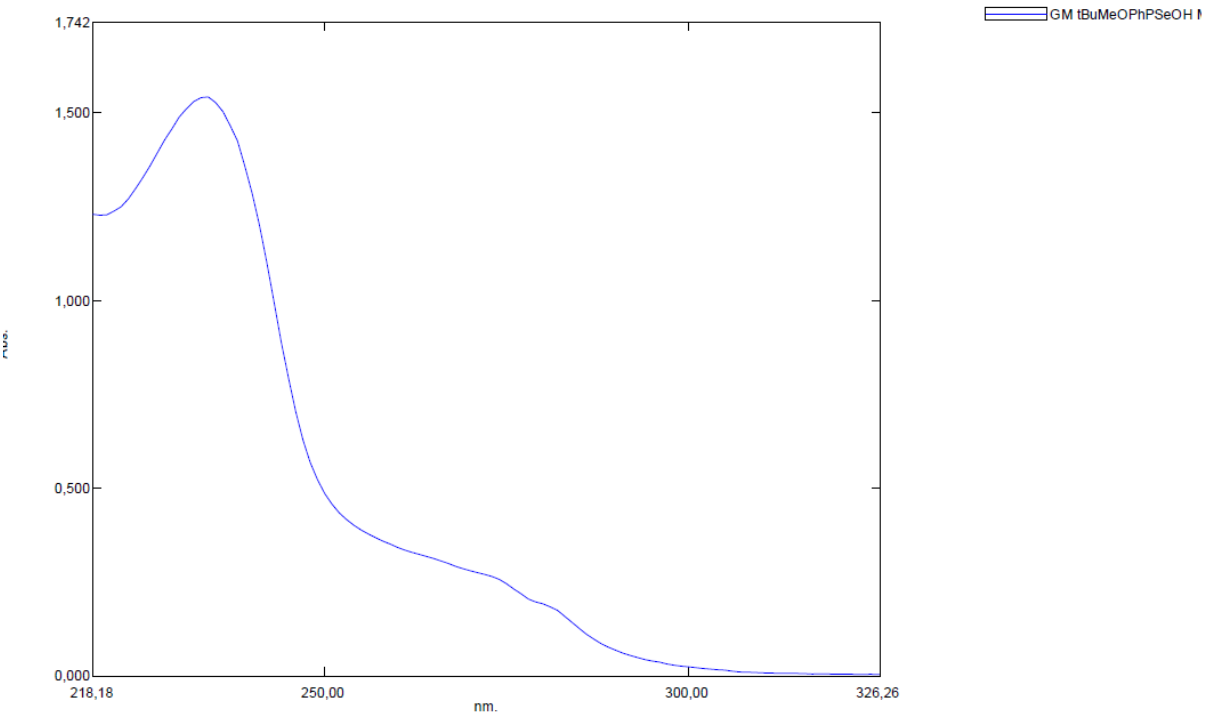

UV-VIS spectrum of *tert*-butyl-(4-trifluoromethyl)phosphinoselenoic acid (2b)

Data Set: GM tBuCF3PhPSeOH MeOH - RawData

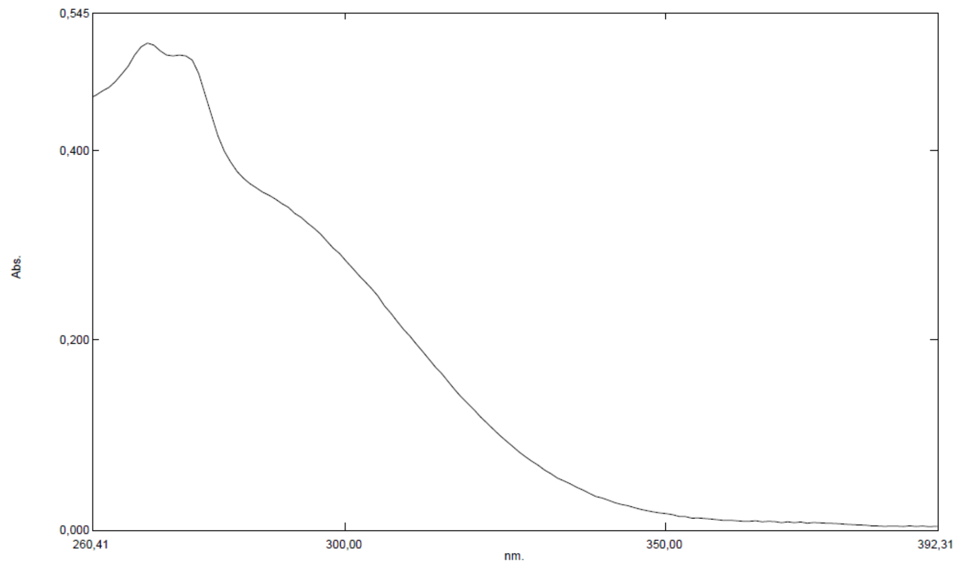

UV-VIS spectrum of *tert*-butyl-1-naphtylphosphinoselenoic acid (2d)

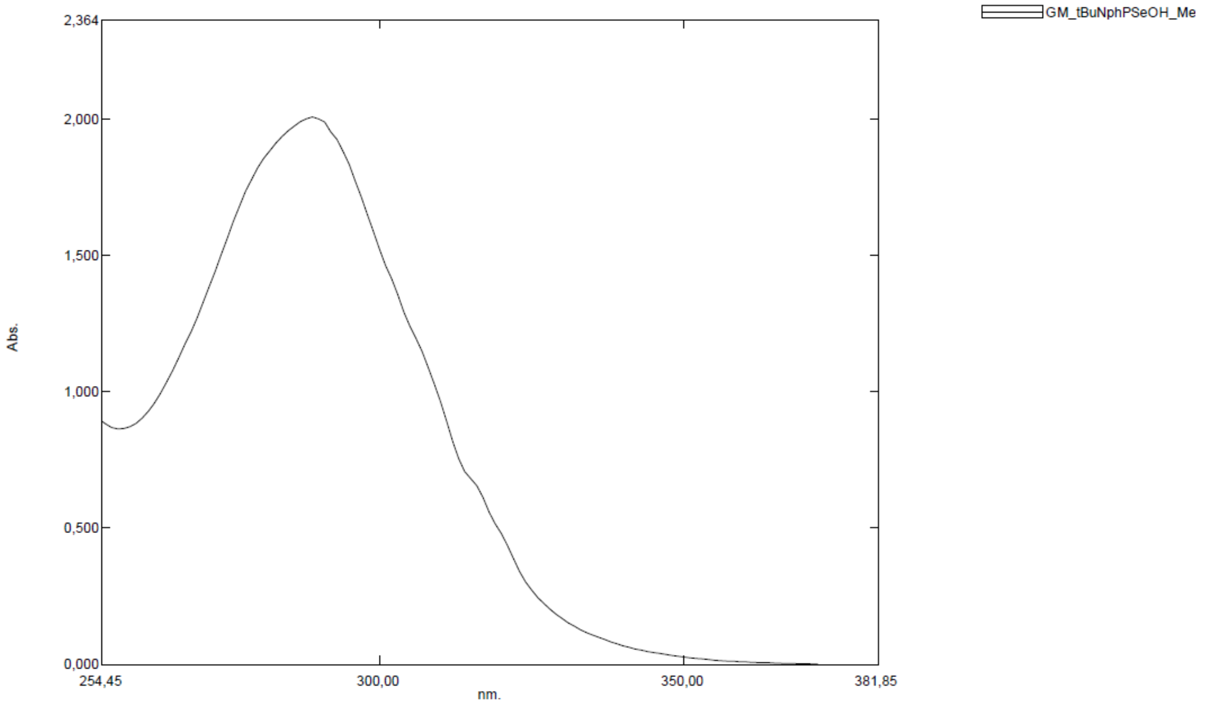

S4. IR Spectra

IR spectrum of *tert*-butyl-(4-methoxyphenyl)phosphinothioic acid (1a)

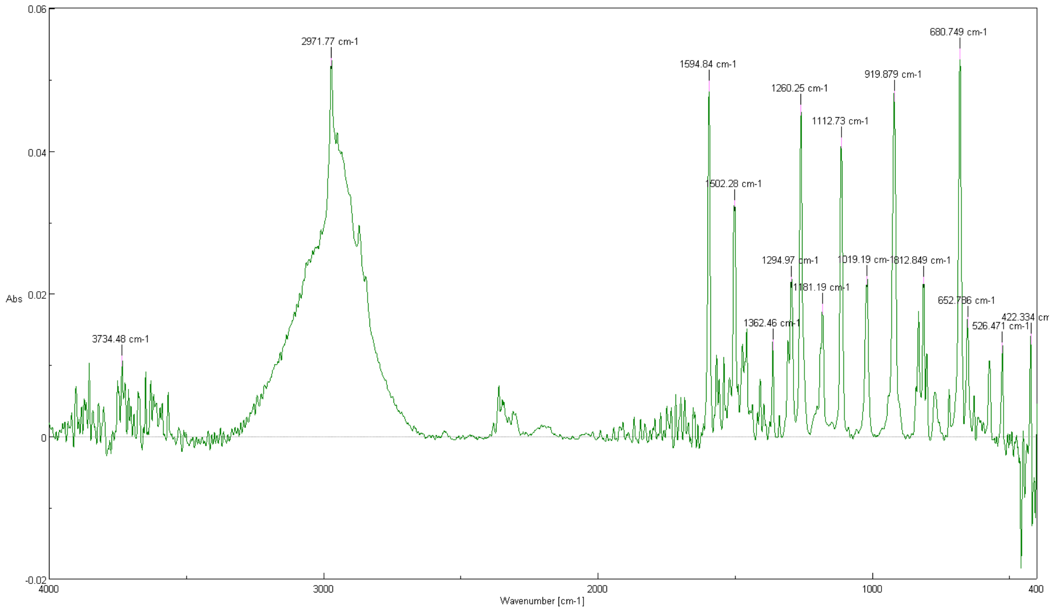

IR spectrum of *tert*-butyl-(4-trifluoromethyl)phosphinothioic acid (1b)

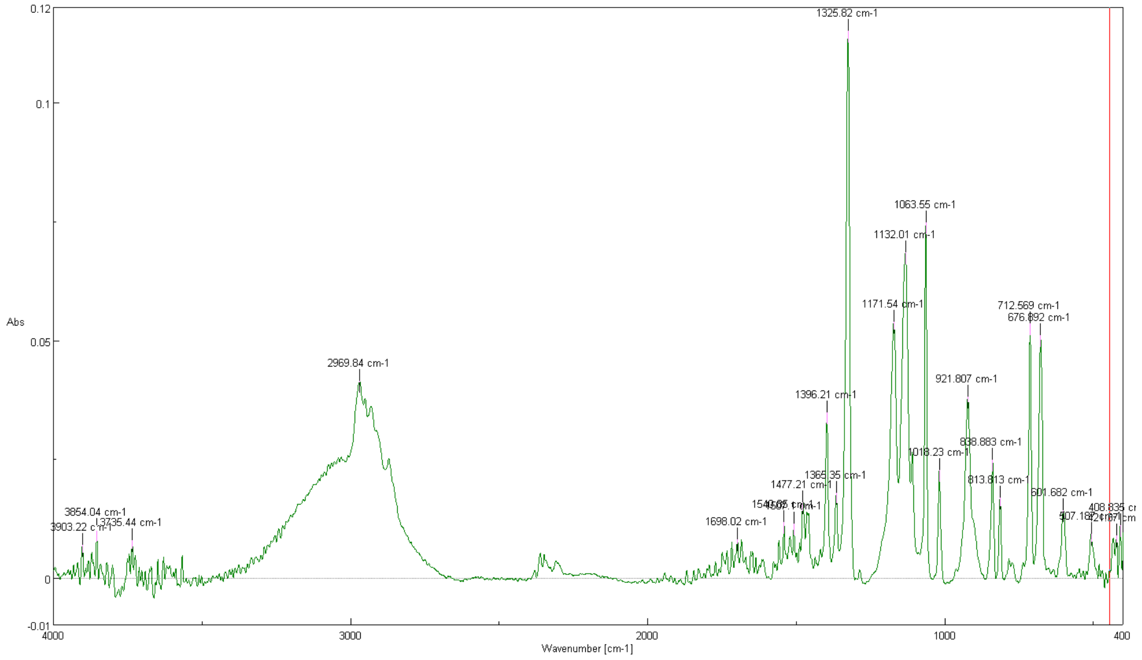

IR spectrum of *tert*-butyl-1-naphtylphosphinothioic acid (1d)

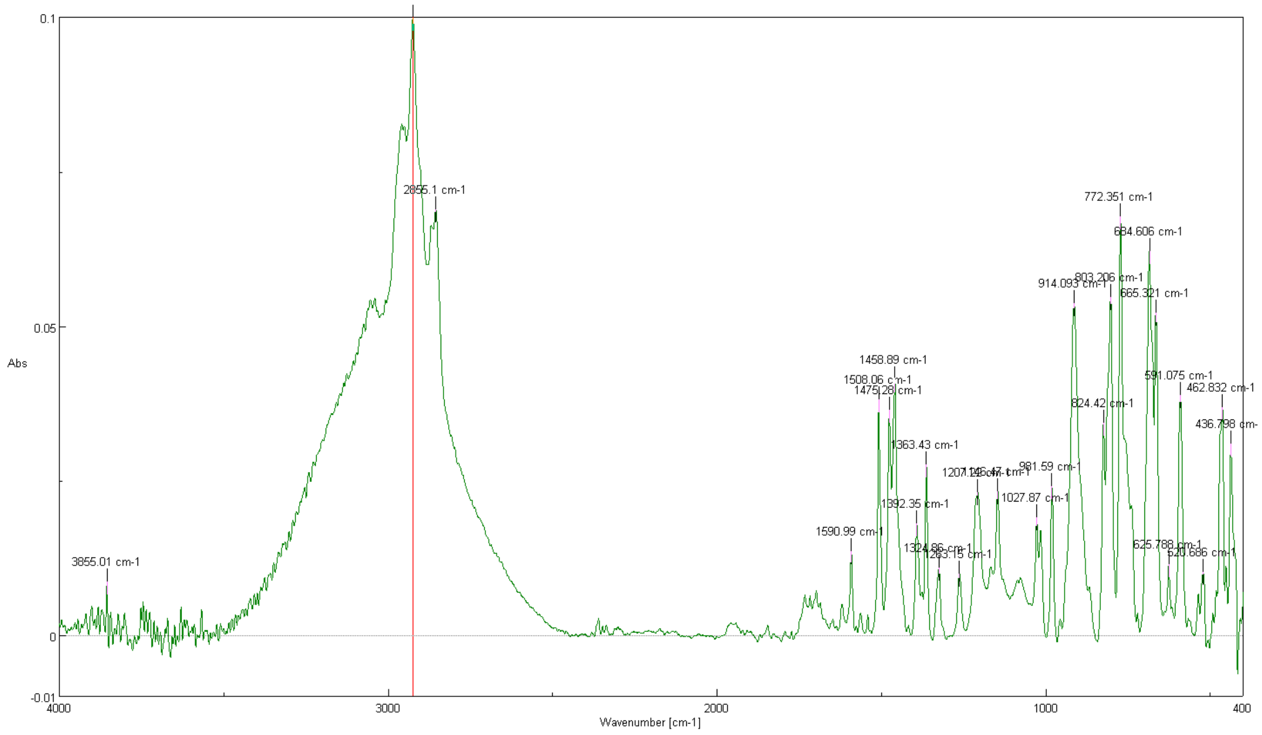

IR spectrum of *tert*-butyl-(4-methoxyphenyl)phosphinoselenoic acid (2a)

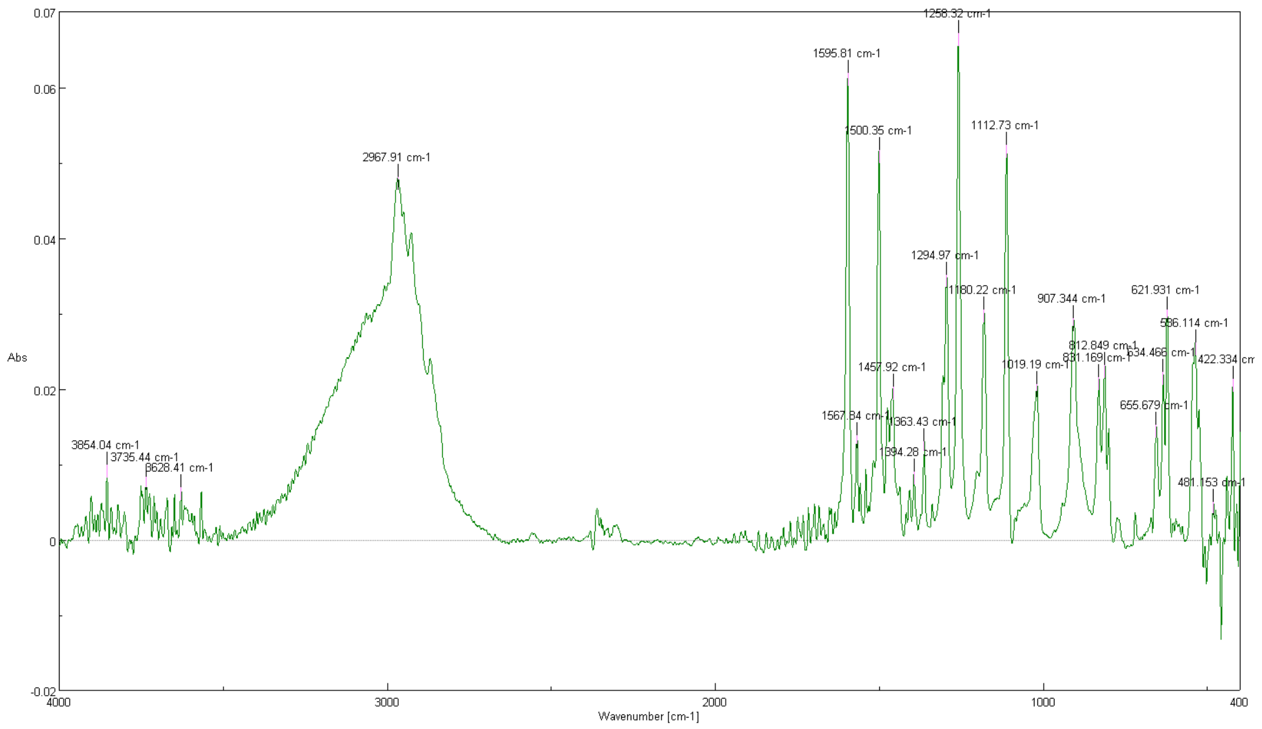

IR spectrum of *tert*-butyl-(4-trifluoromethyl)phosphinoselenoic acid (2b)

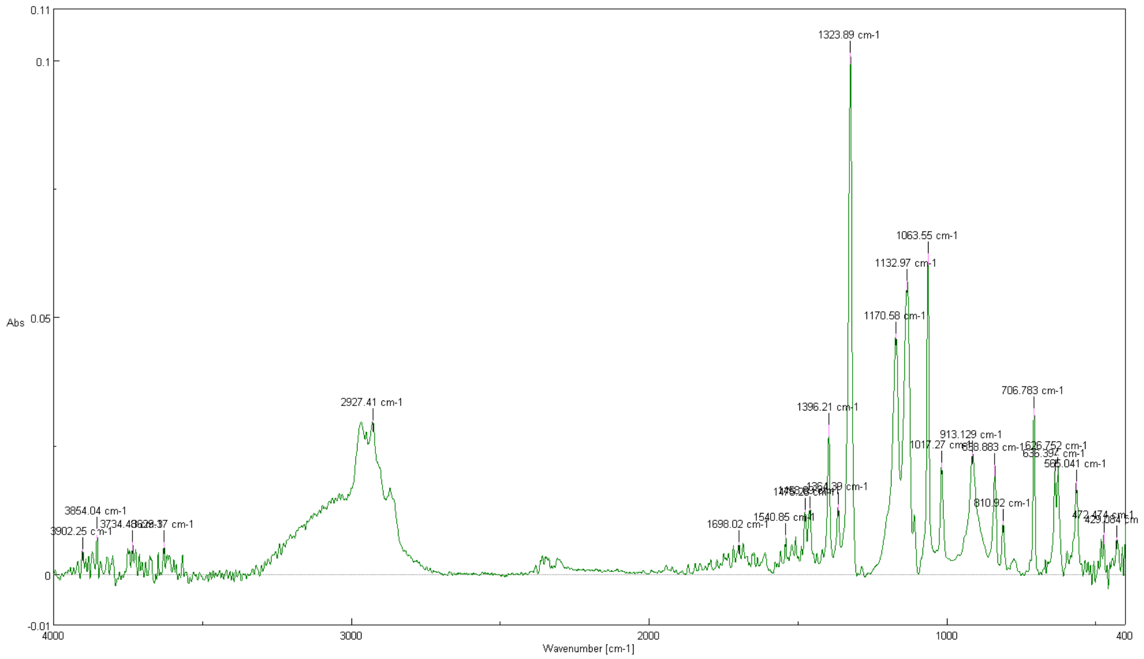

IR spectrum of *tert*-butyl-1-naphtylphosphinoselenoic acid (2d)

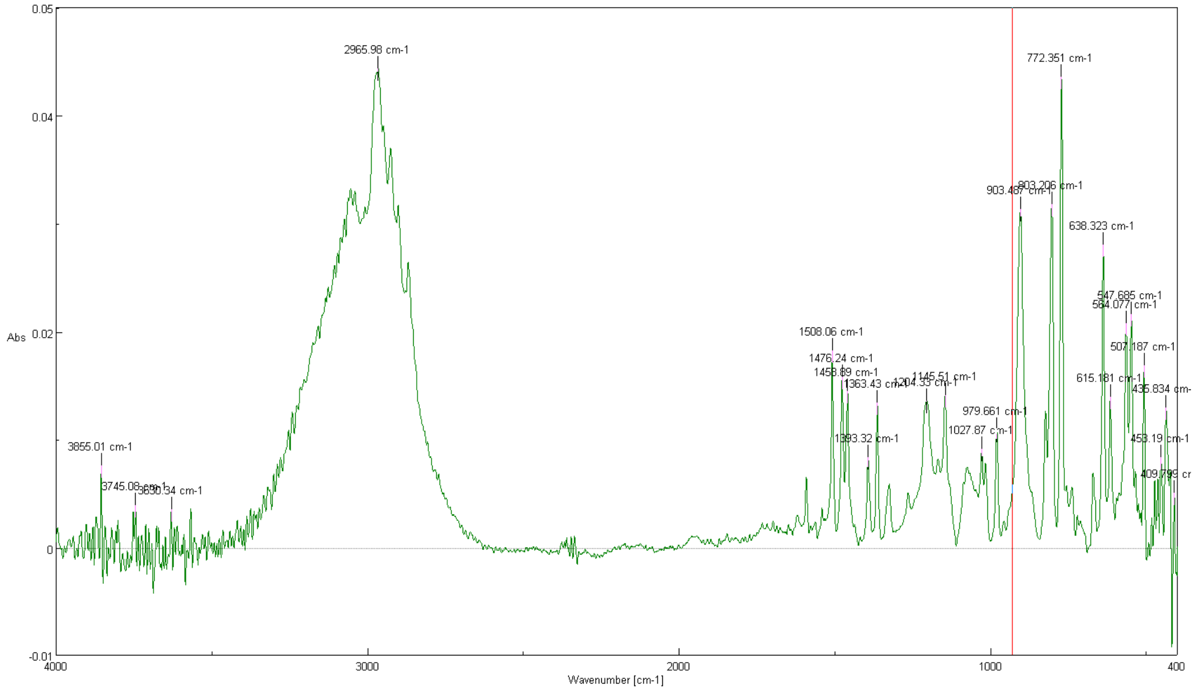

S5. Crystal data and experimental details

Table S1. Crystal data and experimental details of determined phosphinothioic acids **1a-1b.**

***tert*-butyl-(4-methoxyphenyl)phosphinothioic acid (1a)**

Compound		(Sp)-**1a**	(Rp)-**1a**	(rac)-**1a**
CCDC access codes		1509139	1589363	1589364
Empirical formula		C_{11} H17 O2 P S	C11 H17 O2 P S	C11 H17 O2 P S
Formula weight		244.27	244.27	244.27
Temperature (K)		100	100	100
Crystal system		monoclinic	monoclinic	monoclinic
Space group		C2	C2	C2/c
a, (Å)		20.2663(4)	20.2721(2)	20.2613(4)
b, (Å)		11.1526(2)	11.1592(1)	11.0543(2)
c, (Å)		11.4103(2)	11.4136(1)	11.4301(2)
α, (°)		90	90	90
β, (°)		107.193(2)	107.197(1)	106.065(2)
γ, (°)		90	90	90
Volume (Å3)		2463.76(9)	2466.56(4)	2460.08(8)
Z		8	8	8
ρcalc (g/cm^3)		1.317	1.316	1.319
μ (mm^{-1})		3.396	3.392	1.319
F(000)		1040	1040	1040
Radiation		CuKα, λ=1.54178 Å	CuKα, λ=1.54178 Å	CuKα, λ=1.54184 Å
Index ranges h		-24 ≤ h ≤ 23	-23 ≤ h ≤ 24	-24 ≤ h ≤ 23
Index ranges k		-12 ≤ k ≤ 13	-13 ≤ k ≤ 13	-13 ≤ k ≤ 13
Index ranges l		-13 ≤ l ≤ 13	-13 ≤ l ≤ 10	-10 ≤ l ≤ 13
Theta min / max		4.055 / 66.580	4.054 / 66.588	4.542 / 66.596
Reflections:	collected	19191	12747	2899
	independent	4264	4260	2175
Parameters refined		287	284	163
Goodness-of-fit on F2		1.041	1.015	1.148
R1 [I≥2σ(I)]		0.0182	0.0219	0.0322
wR2 [I≥2σ(I)]		0.0182	0.0218	0.0320
R1 [all data]		0.0494	0.0588	0.0771
wR2 [all data]		0.0494	0.0587	0.0770
Flack x		0.000(4)	0.043(7)	racemate
Largest diff. peak (eÅ^{-3})		0.377	0.288	0.417
Largest diff. hole		-0.206	-0.212	-0.288

***tert*-butyl-(4-trifluoromethylphenyl)phosphinothioic acid (1b)**

Compound	(Rp)-**1b**	(rac)-**1b**	bis[(Sp)-**1a**]
CCDC accession codes	2123210	2123234	1589336
Empirical formula	$C_{11}H_{14}F_3OPS$	$C_{11}H_{14}F_3OPS$	$C_{22}H_{32}O_4P_2S_2$
Formula weight	282.25	282.25	486.53
Temperature (K)	100	100	100
Crystal system	orthorhombic	monoclinic	orthorhombic
Space group	P212121	C2/c	P212121
a, (Å)	8.8565(1)	21.884(4)	11.1265(3)
b, (Å)	14.1960(1)	9.104(2)	11.9870(3)
c, (Å)	20.9497(1)	13.752(3)	18.6801(4)
α, (°)	90	90	90
β, (°)	90	108.77(3)	90

γ, (°)		90	90	90
Volume (Å3)		2633.94(4)	2594.2(10)	2491.43(11)
Z		8	8	4
ρcalc (g/cm^3)		1.424	1.445	1.297
μ (mm-1)		3.537	0.390	0.367
F(000)		1168	1168	1032
Radiation		CuKα, λ=1.54178 Å	CuKα, λ=1.54178 Å	CuKα, λ=1.54178 Å
Index ranges h		-10 ≤ h ≤ 10	-24 ≤ h ≤ 26	-11 ≤ h ≤ 13
Index ranges k		-16 ≤ k ≤ 16	-10 ≤ k ≤ 10	-14 ≤ k ≤ 14
Index ranges l		-23 ≤ l ≤ 24	-16 ≤ l ≤ 12	-22 ≤ l ≤ 22
Theta min / max		3.761 / 66.555	1.966 / 25.026	3.316 / 25.021
Reflections:	collected	32249	13316	17487
	independent	4645	2289	4398
Parameters refined		318	174	279
Goodness-of-fit on F2		1.053	1.062	1.041
R1 [all data]		0.0217	0.0286	0.0218
R1 [I≥2σ(I)]		0.0216	0.0282	0.0212
wR2 [all data]		0.0569	0.0751	0.0545
wR2 [I≥2σ(I)]		0.0568	0.0748	0.0542
Flack x		-0.004(6)	racemate	-0.01(3)
Largest diff. peak (eÅ^{-3})		0.249	0.399	0.248
Largest diff. hole		-0.263	-0.233	-0.162

Table S2. Crystal data and experimental details of determined phosphinoselenoic acid **2a.**

***tert*-butyl-(4-methoxyphenyl)phosphinoselenoic acid (2a)**

Compound		(*S*p)-**2a**	(*R*p)-**2a**	(rac)-**2a**
CCDC accession codes		2123219	1509140	2123223
Empirical formula		$C_{11}H_{17}O_2PSe$	$C_{11}H_{17}O_2PSe$	$C_{11}H_{17}O_2PSe$
Formula weight		291.17	291.17	291.17
Temperature (K)		100	100	100
Crystal system		monoclinic	monoclinic	monoclinic
Space group		C2	C2	C2/c
a, (Å)		20.4373(5)	20.4217(5)	20.3696(4)
b, (Å)		11.2841(3)	11.2830(3)	11.0645(2)
c, (Å)		11.6237(3)	11.6107(3)	11.6187(2)
α, (°)		90	90	90
β, (°)		107.842(3)	107.860(1)	105.487(2)
γ, (°)		90	90	90
Volume (Å^3)		2551.69(12)	2546.39(11)	2523.54(8)
Z		8	8	8
ρcalc (g/cm^3)		1.516	1.519	1.533
μ (mm^{-1})		3.048	5.023	3.082
F(000)		1184	1184	1184
Radiation		CuKα, 1.54178 Å	CuKα, 1.54178 Å	CuKα, 1.54178 Å
Index ranges h		-24 ≤ h ≤ 24	-22 ≤ h ≤ 24	-24 ≤ h ≤ 24
Index ranges k		-13 ≤ k ≤ 13	-13 ≤ k ≤ 11	-13 ≤ k ≤ 13
Index ranges l		-13 ≤ l ≤ 13	-13 ≤ k ≤ 10	-13 ≤ k ≤ 13
Theta min / max		3.602 / 25.023	4.000 / 66.584	3.618 / 25.025
Reflections:	collected	14891	11353	21738
	independent	4492	4037	2224
Parameters refined		283	283	157
Goodness-of-fit on F2		1.035	1.057	1.133
R1 [all data]		0.0198	0.0193	0.0244
R1 [I≥2σ(I)]		0.0188	0.0193	0.0244

wR2 [all data]	0.0464	0.0504	0.0518
wR2 [I≥2σ(I)]	0.0462	0.0504	0.0512
Flack x	0.002(4)	-0,002(15)	racemate
Largest diff. peak (eÅ^{-3})	0.583	0.302	0.445
Largest diff. hole	-0.242	-0.221	-0.251
